# Suvemcitug plus chemotherapy in women with platinum-resistant recurrent ovarian cancer: the SCORES randomized, double-blinded, phase 3 trial

**DOI:** 10.1038/s43018-025-01085-z

**Published:** 2026-01-09

**Authors:** Guangwen Yuan, Ge Lou, Jundong Li, Mei Xu, Xiaowei Liu, Danbo Wang, Keqiang Zhang, Tao Zhu, Xiumin Li, Yi Huang, Wei Duan, Ke Wang, Qi Zhou, Guiling Li, Chen Yang, Jiajing Zhang, Haolin Sun, Renhong Tang, Qingshui Li, Lingying Wu

**Affiliations:** 1 Department of Gynecology Oncology, National Cancer Center/National Clinical Research Center for Cancer/Cancer Hospital, Chinese Academy of Medical Sciences and Peking Union Medical College, Beijing, China; 2 Department of Gynecology, Harbin Medical University Cancer Hospital, Harbin, China; 3 Department of Gynecology, Sun Yat-Sen University Cancer Center, Guangzhou, China; 4 Department of Obstetrics and Gynecology, Xuzhou Central Hospital, Xuzhou, China; 5 Department of Oncology, Affiliated Hospital of Jining Medical University, Jining, China; 6 Department of Gynecology, Liaoning Cancer Hospital and Institute, Shenyang, China; 7 Department of Gynecologic Oncology, Hunan Cancer Hospital, Changsha, China; 8 Department of Gynecologic Oncology, Zhejiang Cancer Hospital, Hangzhou, China; 9 Department of Gynecologic Oncology, Linyi Cancer Hospital, Linyi, China; 10 Department of Gynecologic Oncology, Hubei Cancer Hospital, Wuhan, China; 11 Department of Gynecologic Oncology, Beijing Obstetrics and Gynecology Hospital, Capital Medical University, Beijing, China; 12 Department of Gynecologic Oncology, Tianjin Medical University Cancer Institute & Hospital, Tianjin, China; 13 Department of Gynecologic Oncology, Chongqing Cancer Hospital, Chongqing, China; 14 Department of Gynecologic Oncology, Union Hospital, Tongji Medical College, Huazhong University of Science and Technology, Wuhan, China; 15 Clinical Sciences, Simcere Zaiming Pharmaceutical Co., Ltd., Shanghai, China; 16 State Key Laboratory of Neurology and Oncology Drug Development, Nanjing, China; 17 Clinical Statistics, Simcere Zaiming Pharmaceutical Co., Ltd., Shanghai, China; 18 Simcere Zaiming Pharmaceutical Co., Ltd., Shanghai, China; 19 Department of Gynecologic Oncology, Shandong Cancer Hospital and Institute, Shandong First Medical University and Shandong Academy of Medical Sciences, Jinan, China; 20 National Cancer Center/National Clinical Research Center for Cancer/Cancer Hospital & Liaoning Hospital, Chinese Academy of Medical Sciences and Peking Union Medical College, Shenyang, China

**Keywords:** Cancer, Gynaecological cancer, Cancer therapy, Gynaecological cancer, Clinical trials

## Abstract

In the SCORES study (NCT04908787), women with ovarian cancer that progressed within 6 months after completing platinum-based therapy were randomized (2:1) to receive suvemcitug (1.5 mg kg^−1^), an antibody to vascular endothelial growth factor or placebo every 2 weeks, with chemotherapy (paclitaxel, topotecan or PEGylated liposomal doxorubicin). The primary endpoint was progression-free survival (PFS). The key secondary endpoint was overall survival (OS). Other secondary endpoints included objective response rate, disease control rate, duration of response, quality of life, safety, pharmacokinetics and antidrug antibodies. Between June 5, 2021 and October 11, 2024, 421 participants were randomized (49.4% and 49.4% previously exposed to antiangiogenic agents and poly(ADP-ribose) polymerase inhibitors, respectively). Median PFS was 5.5 and 2.7 months in the suvemcitug and placebo arms, respectively (hazard ratio: 0.46, 95% confidence interval (CI): 0.35–0.60, *P* < 0.001), meeting the primary endpoint. Median OS was 15.3 versus 14.0 months, respectively (hazard ratio: 0.77, 95% CI: 0.60–0.99, *P* = 0.03). Decreased neutrophil count and decreased white blood cell count were the most common grade ≥3 treatment-emergent adverse events (TEAEs) in the suvemcitug arm. No suvemcitug-related grade 5 TEAE occurred. In conclusion, the addition of suvemcitug to chemotherapy significantly improved PFS and OS, with tolerable toxicities.

## Abstract (web summary)

Wu and colleagues present the efficacy and safety results from the phase 3 SCORES trial evaluating suvemcitug plus chemotherapy in persons with platinum-refractory or resistant recurrent ovarian cancer.

## Main

Ovarian cancer (OC) is the most lethal gynecological malignancy, with 324,938 new cases and 206,834 deaths in 2022 globally^1^. Platinum-based chemotherapy plus paclitaxel with or without bevacizumab, recently with maintenance poly(ADP-ribose) polymerase (PARP) inhibitors and/or bevacizumab, is the primary treatment option for advanced OC^2–6^. Despite a 75–80% response rate with first-line therapy, relapse occurs within 18 months in the majority of persons^7,8^. Standard nonplatinum chemotherapy for platinum-resistant OC has limited efficacy, with ≤15% of persons showing an objective response and a median progression-free survival (PFS) between 3 and 4 months^7,9,10^.

Bevacizumab, a monoclonal antibody to vascular endothelial growth factor (VEGF), has demonstrated efficacy for both platinum-sensitive and resistant OC^11,12^. In the AURELIA trial, bevacizumab, when added to chemotherapy, extended the median PFS by 3.3 months in participants with platinum-resistant OC^13^. On the basis of these findings, bevacizumab is recommended for the treatment of persons with platinum-resistant OC who have received ≤2 prior lines of cytotoxic therapy^14^. The efficacy of bevacizumab, however, needs to be reexamined as persons who received PARP inhibitors were not included. Furthermore, the AURELIA trial only included participants who received ≤2 prior lines of cytotoxic therapy and only 7.2% of the participants received prior antiangiogenic therapy.

Antiangiogenic agents other than bevacizumab, including ofranergene obadenovec, failed to improve objective response and survival in persons with platinum-resistant OC when added to chemotherapy^8,15^. Novel safe and effective antiangiogenic drugs are urgently needed for persons with platinum-resistant OC.

Suvemcitug (BD0801), a humanized rabbit monoclonal IgG1 (*κ*) anti-VEGF antibody, selectively binds to and prevents VEGF-A from binding to VEGF receptors 1 and 2 (VEGFR1 and VEGFR2)^16,17^. VEGF-A is secreted in multiple forms by alternative splicing^18^; these include VEGF_121_, VEGF_165_ and VEGF_189_. Suvemcitug and bevacizumab have comparable binding affinity for VEGF_121_ and VEGF_189_ (ref. ^19^). Suvemcitug and bevacizumab bind to different epitopes of human VEGF_165_ (ref. ^17^) but have comparable affinity for VEGF_165_ (*K*_d_: 1.2 × 10^−11^ M versus 1.0 × 10^−11^ M; half-maximal effective concentration: 7.0 ng ml^−1^ versus 5.8 ng ml^−1^). Suvemcitug also binds to VEGF_164_ with an affinity similar to VEGF_165_, whereas bevacizumab does not bind to VEGF_164_. In comparison to bevacizumab, suvemcitug has a lower half-maximal inhibitory concentration for inhibition of VEGF binding to VEGFR1 (21.0 ng ml^−1^ versus 6760 ng ml^−1^) and VEGFR2 (275.4 ng ml^−1^ versus 1451 ng ml^−1^)^19^. Early-stage trials of suvemcitug have shown promising antitumor activities when used in combination with chemotherapy for previously treated advanced solid tumors^19^. A phase 1b trial of suvemcitug plus paclitaxel or topotecan reported objective response in nine of 29 participants (31%) with platinum-resistant OC and a median PFS of 5.4 months^20^. In these trials, the safety profile of suvemcitug was manageable without unexpected toxicities.

We conducted a phase 3 trial (SCORES) to examine the efficacy and safety of suvemcitug plus chemotherapy in persons with platinum-refractory or resistant OC.

## Results

### Participants

**Fig. 1:**
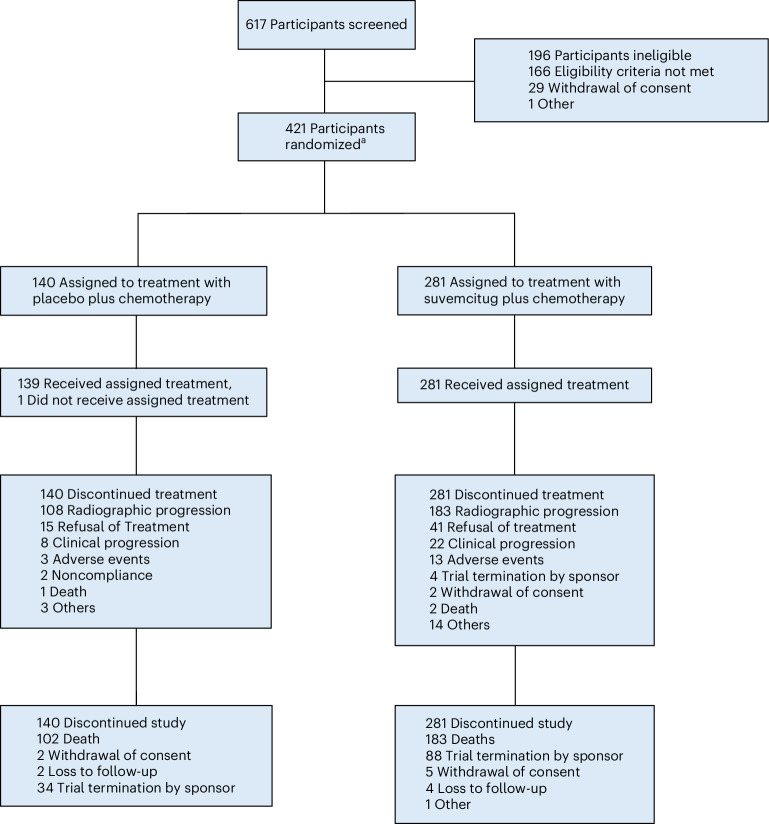
Participant flow in the SCORES trial. ^a^Participants were randomized in a 2:1 ratio and stratified according to platinum-refractory status (yes versus no), number of prior systemic therapies (one versus two), chemotherapeutic agent (paclitaxel versus PEGylated liposomal doxorubicin versus topotecan) and prior antiangiogenic therapy (yes versus no). More information is provided in the Table 1 footnotes. For the safety analysis, one participant who was randomized but did not receive planned treatment was excluded.

This randomized, double-blind, placebo-controlled, phase 3 trial (SCORES) was conducted at 55 tertiary-care centers in China between June 5, 2021 and October 11, 2024. Randomization was stratified according to platinum-refractory status (yes versus no), number of prior systemic therapies (one versus two), chemotherapeutic agent (paclitaxel versus PEGylated liposomal doxorubicin versus topotecan) and prior antiangiogenic therapy (yes versus no). A total of 617 women (aged ≥18 years) with histologically confirmed epithelial ovarian, fallopian tube or primary peritoneal cancer were screened for eligibility. Participants were required to have platinum-refractory or resistant disease (disease progression within 6 months of platinum therapy), at least one measurable lesion per the response evaluation criteria in solid tumors (RECIST; v.1.1), an Eastern Cooperative Oncology Group (ECOG) performance status of 0–1 and adequate hematologic and organ function. In total, 421 eligible participants were randomized (2:1) to receive suvemcitug (1.5 mg kg^−1^ infused on days 1 and 15 of each 4-week cycle) plus chemotherapy (suvemcitug arm; *n* = 281) or placebo plus chemotherapy (placebo arm; *n* = 140) (Fig. 1). Most participants (414, 98.3%) had high-grade serous adenocarcinoma and 383 (91.0%) had International Federation of Gynecology and Obstetrics (FIGO) stage III or IV disease. The majority of the participants (294, 69.8%) received ≥2 prior lines of systemic therapy and 208 (49.4%) had previous exposure to an antiangiogenic agent (bevacizumab: 184, 43.7%) and a PARP inhibitor. Demographic and baseline characteristics of the participants are shown in Table 1.

**Table 1:** Demographic and baseline characteristics of the participants in the full analysis set

	Suvemcitug plus chemotherapy (*n* = 281)	Placebo plus chemotherapy (*n* = 140)	*P* value^#^
**Age, years**			0.9999
** Median age (range)**	56.0 (36–76)	55.0 (34–79)	
** >65**	38 (13.5)	20 (14.3)	
**Ethnic groups**			0.873
** Han Chinese**	266 (94.7)	132 (94.3)	
** Other**	15 (5.3)	8 (5.7)	
**Origin of cancer**			0.014
** Epithelial OC**	263 (93.6)	120 (85.7)	
** Fallopian tube cancer**	15 (5.3)	19 (13.6)	
** Primary peritoneal cancer**	3 (1.1)	1 (0.7)	
**ECOG performance status score**^**†**^			0.063
** 0**	106 (37.7)	40 (28.6)	
** 1**	175 (62.3)	100 (71.4)	
**Histologic diagnosis**			0.176
** High-grade serous adenocarcinoma**	278 (98.9)	136 (97.1)	
** Endometrioid carcinoma**	3 (1.1)	4 (2.9)	
** Sum of the target lesion diameter, median (range), mm**	41.20 (10.0–322.8)	43.05 (10.7–229.1)	0.495
**FIGO stage**			0.901
** I**	7 (2.5)	4 (2.9)	
** II**	13 (4.6)	9 (6.4)	
** III**	195 (69.4)	95 (67.9)	
** IV**	62 (22.1)	31 (22.1)	
** Unknown**	4 (1.4)	1 (0.7)	
**Confirmed distant metastasis**			
** Peritoneum**	160 (56.9)	79 (56.4)	0.923
** Lymph node**	181 (64.4)	78 (55.7)	0.084
** Pelvic cavity**	98 (34.9)	53 (37.9)	0.548
** Liver**	86 (30.6)	39 (27.9)	0.561
** Lungs**	22 (7.8)	16 (11.4)	0.225
** Spleen**	26 (9.3)	13 (9.3)	0.991
** Bone**	9 (3.2)	4 (2.9)	0.847
** Kidney**	0	2 (1.4)	0.045
** Other**	151 (53.7)	81 (57.9)	0.423
**Previous lines of systemic therapy**			0.812
** 1**	87 (31.0)	40 (28.6)	
** 2**	106 (37.7)	52 (37.1)	
** 3**	59 (21.0)	35 (25.0)	
** ≥4**	29 (10.3)	13 (9.3)	
**Platinum-free interval, months**			0.369
** <1**	29 (10.3)	21 (15.0)	
** 1–3**	81 (28.8)	37 (26.4)	
** ≥3**	171 (60.9)	82 (58.6)	
**Previous chemotherapy**			
** Platinum-based drugs**	281 (100)	140 (100)	-
** Taxanes**	279 (99.3)	138 (98.6)	0.475
** Anthracyclines**	71 (25.3)	23 (16.4)	0.040
** Topoisomerase 1 inhibitors**	4 (1.4)	2 (1.4)	0.997
** Other**	49 (17.4)	24 (17.1)	0.940
**Chemotherapeutic agents**			0.998
** Paclitaxel**	124 (44.1)	62 (44.3)	
** PEGylated liposomal doxorubicin**	88 (31.3)	44 (31.4)	
** Topotecan**	69 (24.6)	34 (24.3)	
**Previous antiangiogenic therapy**			0.972
** Yes**	139 (49.5)	69 (49.3)	
** No**	142 (50.5)	71 (50.7)	
** Previous bevacizumab therapy**	123 (43.8)	61 (43.6)	0.969
** Previous PARP inhibitor therapy**	138 (49.1)	70 (50.0)	0.863
**Ascites**			0.981
** Yes**	92 (32.7)	46 (32.9)	
** No**	189 (67.3)	94 (67.1)	
**Pleural effusion**			0.634
** Yes**	26 (9.3)	11 (7.9)	
** No**	255 (90.7)	129 (92.1)	
**CA-125**			0.597
** ≤2** **×** **ULN**	36 (12.8)	14 (10.0)	
** 2** **×** **ULN–1,000**	178 (63.3)	95 (67.9)	
** >1,000**	67 (23.8)	31 (22.1)	
**Platinum-refractory**			0.713
** No**	256 (91.1)	126 (90.0)	
** Number of prior systemic therapies** ^**‡**^			0.807
** 1**	190 (67.6)	93 (66.4)	
** 2**	91 (32.4)	47 (33.6)	

Data are numbers (%) unless otherwise specified. Percentages may not total 100 because of rounding.

^#^Two-sided chi-square test; no adjustment for multiple comparisons.

^†^ECOG performance status scores are on a scale of 0–5, with higher scores indicating greater disability.

^‡^A value of 1 represents no systemic therapy after platinum resistance; a value of 2 indicates systemic therapy after platinum resistance.

At the final analysis (October 11, 2024), all participants discontinued the treatment, mostly for disease progression (73.0% and 82.9% in the suvemcitug and placebo arms, respectively) (Fig. 1).

### Efficacy

**Fig. 2:**
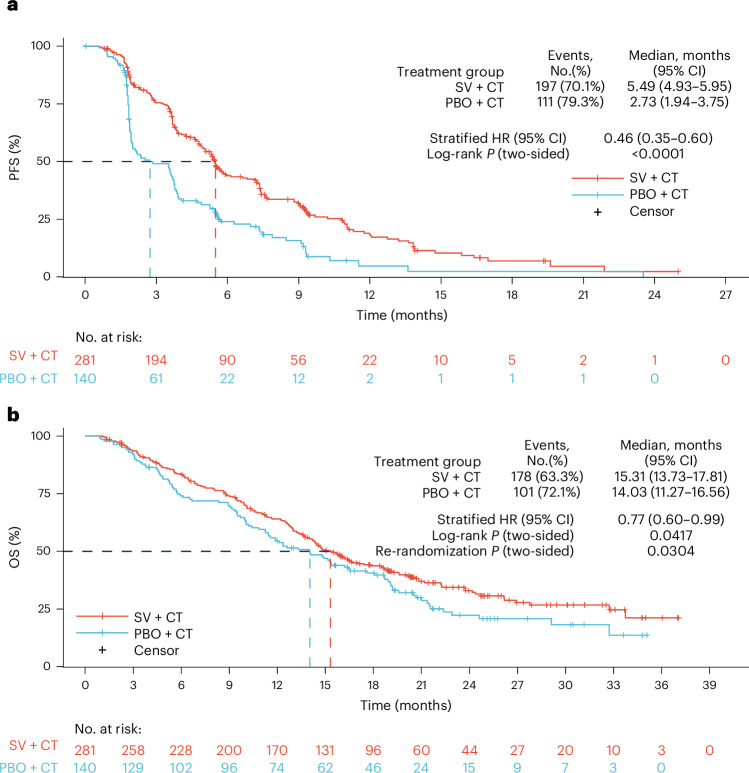
Survival outcomes. **a**, Kaplan–Meier curve of PFS at the first efficacy analysis in the full analysis set assessed by BIRC per RECIST (v.1.1). Number of participants: 281 and 140 in the suvemcitug and placebo arms, respectively. *P* < 0.0001. **b**, Kaplan–Meier curve of OS in the full analysis set at the final analysis. Number of participants: 281 and 140 in the suvemcitug and placebo arms, respectively. *P* = 0.03. PBO + CT, placebo plus chemotherapy; SV + CT, suvemcitug plus chemotherapy. Source data

The primary endpoint was PFS, as assessed by a blinded independent review committee (BIRC) per RECIST (v.1.1) when 308 events had occurred. At the data cutoff (December 8, 2023), the median follow-up duration was 14.4 and 14.3 months in the suvemcitug and placebo arms, respectively. The median PFS was 5.5 months in the suvemcitug arm (95% confidence interval (CI): 4.9–6.0) versus 2.7 months in the placebo arm (95% CI: 1.9–3.8; stratified hazard ratio (HR): 0.46, 95% CI: 0.35–0.60, *P* < 0.001) (Fig. 2a and Supplementary Table 1).

At the final analysis (October 11, 2024), the median follow-up duration was 23.7 and 23.4 months in the suvemcitug and placebo arms, respectively. A total of 178 and 101 overall survival (OS) events occurred in the suvemcitug and placebo arms, respectively. The median OS was 15.3 months (95% CI: 13.7–17.8) in the suvemcitug arm versus 14.0 months (95% CI: 11.3–16.6) in the placebo arm (stratified HR: 0.77, 95% CI: 0.60–0.99, *P* = 0.03) (Fig. 2b).

**Fig. 3:**
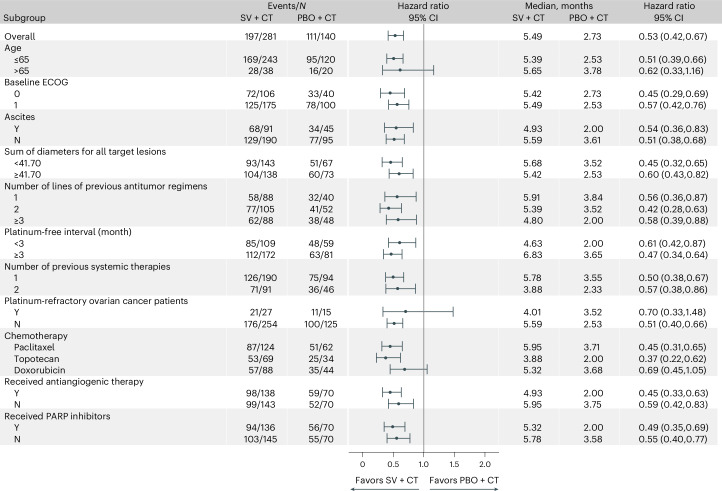
Forest plots for PFS per BIRC. Shown are the results of prespecified and unplanned post hoc analysis for previous received PARP (yes, no) subgroup analyses in the full analysis set. The HR for progression or death was based on Cox proportional-hazards regression analysis for all randomized participants. No stratification was used in the forest plots. Source data

**Extended Data Fig. 1:**
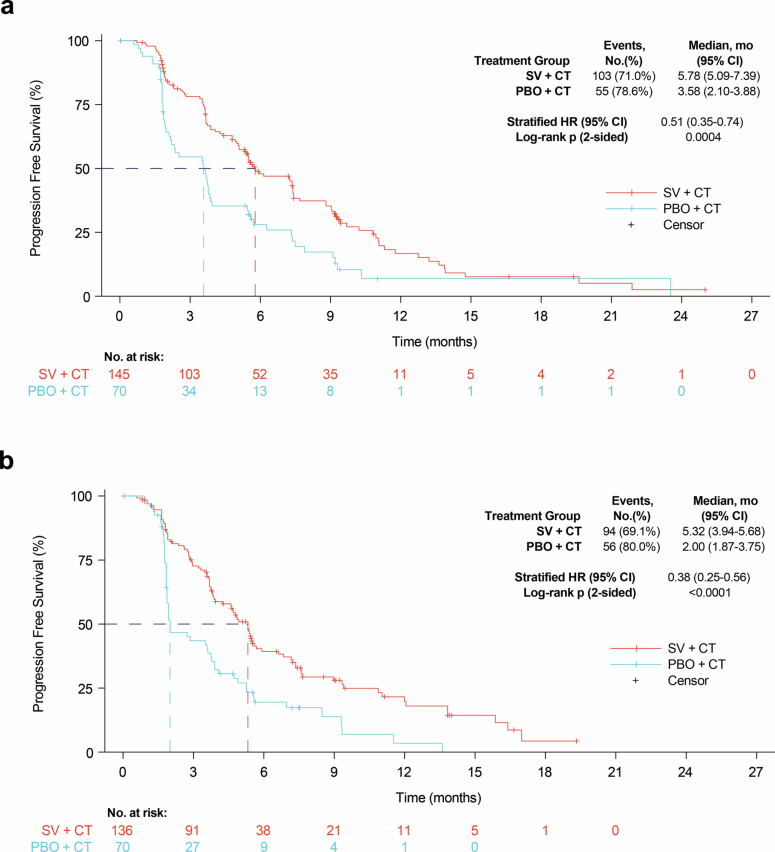
Subgroup analyses of progression-free survival based on prior exposure to PARP inhibitors. **a**, patients who were PARP inhibitor naïve. Number of patients: 145 and 70 in the suvemcitug and placebo groups, respectively. *P* = *0.0004*. **b**, patients who had received prior PARP inhibitor therapy. Number of patients: 136 and 70 in the suvemcitug and placebo groups, respectively. *P* < *0.0001*. Source data

The results of subgroup analyses of BIRC-assessed PFS consistently favored the suvemcitug arm across all prespecified and unplanned post hoc analysis for previous PARP inhibitor exposure (Fig. 3). In the paclitaxel cohort, suvemcitug led to a 2.2-month extension in the median PFS (6.0 months versus 3.7 months in the placebo arm; HR: 0.45, 95% CI: 0.31–0.65). In the topotecan cohort, the median PFS was 3.9 months in the suvemcitug arm versus 2.0 months in the placebo arm (HR: 0.37, 95% CI: 0.22–0.62). In the doxorubicin cohort, the median PFS was 5.3 months in the suvemcitug arm versus 3.7 months in the placebo arm (HR: 0.69, 95% CI: 0.45–1.05). Notably, suvemcitug increased the median PFS regardless of previous exposure to PARP inhibitors (no, HR: 0.55, 95% CI: 0.40–0.77; yes, HR: 0.49, 95% CI: 0.35–0.69) (Fig. 3 and Extended Data Fig. 1a,b) and regardless of previous exposure to antiangiogenic agents (no, HR: 0.59, 95% CI: 0.42–0.83; yes, HR: 0.45, 95% CI: 0.33–0.63) (Fig. 3).

**Extended Data Fig. 2:**
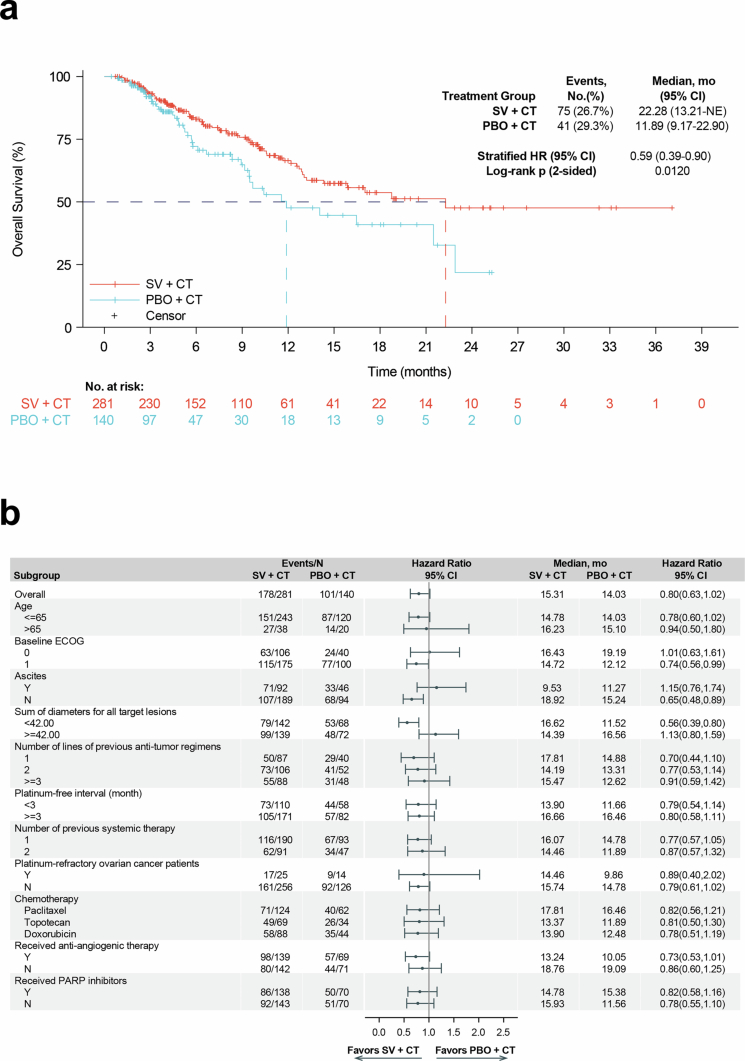
Supplementary and subgroup analyses of overall survival. **a**, Kaplan-Meier curve of overall survival of patients without subsequent antitumor therapy. Number of patients: 281 and 140 in the suvemcitug and placebo groups, respectively. *P* = *0.01*. Hypothetical estimands strategy was used for the supplemental analysis. 95% CI, 95% confidence interval; HR, hazard ratio; PBO + CT, placebo plus chemotherapy; SV + CT, suvemcitug plus chemotherapy. **b**, Forest plots for overall survival. Shown are the results of prespecified subgroup analyses of overall survival at the final analysis in the full analysis set. The hazard ratio (HR) for progression or death that is reported for all the randomized patients was based on a Cox proportional-hazards model. Source data

Supplementary analysis that was undertaken to account for subsequent antitumor therapy as intercurrent events showed that suvemcitug led to a 10.4-month extension of median OS compared to placebo (22.3 months, 95% CI: 13.2–NE (not evaluable) versus 11.9 months, 95% CI: 9.2–22.9; stratified HR: 0.59, 95% CI: 0.39–0.90, *P* = 0.01) (Extended Data Fig. 2a).

Subgroup analyses of OS showed significant reduction in the risk of death across in the suvemcitug arm across almost all prespecified and unplanned post hoc analysis for previous PARP inhibitor exposure (Extended Data Fig. 2b). In participants who were previously treated with anti-VEGF agents, suvemcitug led to a 27% reduction in the risk of death compared to placebo (HR: 0.73, 95% CI: 0.53–1.01). Similar findings were observed in participants previously exposed to PARP inhibitors (HR: 0.82, 95% CI: 0.58–1.16).

**Fig. 4:**
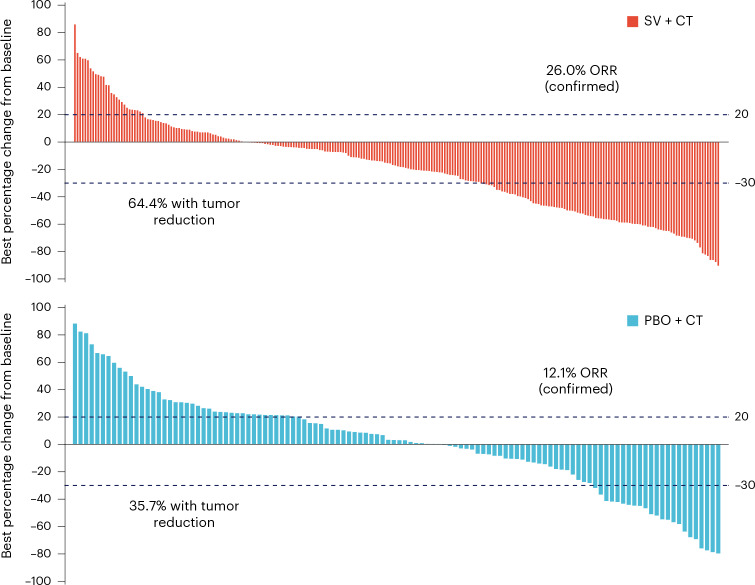
Treatment responses. Waterfall plots of the best percentage changes for the sum of target lesion diameters are shown for individual participants with platinum-refractory or resistant recurrent OC assessed by BIRC per RECIST (v.1.1). Number of participants: 281 and 140 in the suvemcitug and placebo arms, respectively. The lower dashed line indicates a 30% reduction and the upper dashed line represents a 20% increase in the target lesion size. The ORR was defined as the proportion of participants in the full analysis set with a complete response or a partial response. Source data

Objective response at the first analysis was confirmed in 73 of 281 participants (26.0%; 95% CI: 21.0–31.5%) by BIRC in the suvemcitug arm versus 17 of 140 participants (12.1%) in the placebo arm (95% CI: 7.2–18.7%, *P* = 0.001) (Fig. 4). The median duration of response (DOR) was 8.8 months (95% CI: 6.1–10.9) versus 6.1 months (95% CI 4.2–NE). Disease control per BIRC was attained in 215 of 281 participants (76.5%; 95% CI: 71.1–81.3%) in the suvemcitug arm versus 69 of 140 participants (49.3%; 95% CI: 40.7–57.9%) in the placebo arm (*P* < 0.001) (Supplementary Table 1).

### Safety

At the final analysis, the median duration of treatment was 18.9 weeks and 9.1 weeks for suvemcitug and placebo, respectively. One participant in the placebo arm was not treated and excluded from safety analysis. The mean relative dose intensities of paclitaxel, PEGylated liposomal doxorubicin or topotecan were slightly lower in the suvemcitug arm than the placebo arm (Supplementary Table 2).

Treatment-emergent adverse effects (TEAEs) of any grade occurred in 281 participants (100%; grade ≥3: 234 participants, 83.3%) in the suvemcitug arm and 137 participants (98.6%; grade ≥3: 92, 66.2%) in the placebo arm (Table 2). The most frequently reported grade ≥3 TEAEs (occurring in ≥15% of the participants in either arm) included neutrophil count decreased (suvemcitug: 49.8%, 140/281 versus placebo: 41.0%, 57/139), white blood cell count decreased (suvemcitug: 35.9%, 101/281 versus placebo: 27.3%, 38/139), hypertension (suvemcitug: 18.9%, 53/281 versus placebo: 0.7%, 1/139) and anemia (suvemcitug: 16.7%, 47/281 versus placebo: 17.3%, 24/139) (Table 2). One participant (0.4%) in the suvemcitug arm had gastrointestinal perforation versus none in the placebo arm. TEAEs led to suvemcitug dose reduction in 26 participants (9.3%) and placebo dose reduction in none of the participants in the placebo arm. TEAEs led to suvemcitug treatment interruption in 232 participants (82.6%) and placebo treatment interruption in 86 participants (61.9%). Suvemcitug treatment was discontinued in 19 participants (6.8%) and placebo treatment was discontinued in three (2.2%) participants (Table 2).

**Table 2:** Incidence of TEAEs occurring in at least 10% of the participants in either arm of the safety population at the final analysis

	Suvemcitug plus chemotherapy (*n* = 281)		Placebo plus chemotherapy (*n* = 139)		*P* value*	
**Preferred term**	All grades	Grade ≥3	All grades	Grade ≥3	All grades	Grade ≥3
**Any**	281 (100)	234 (83.3)	137 (98.6)	92 (66.2)	0.044	<0.001
**Neutrophil count decreased**	238 (84.7)	140 (49.8)	94 (67.6)	57 (41.0)	<0.001	0.089
**White blood cell count decreased**	237 (84.3)	101 (35.9)	102 (73.4)	38 (27.3)	0.007	0.078
**Anemia**	201 (71.5)	47 (16.7)	111 (79.9)	24 (17.3)	0.066	0.890
**Platelet count decreased**	143 (50.9)	34 (12.1)	43 (30.9)	10 (7.2)	0.001	0.122
**Proteinuria**	112 (39.9)	11 (3.9)	19 (13.7)	0	<0.001	0.018
**Asthenia**	97 (34.5)	4 (1.4)	34 (24.5)	4 (2.9)	0.036	0.305
**Alanine aminotransferase increased**	83 (29.5)	1 (0.4)	24 (17.3)	0	0.007	0.481
**Aspartate aminotransferase increased**	81 (28.8)	0	26 (18.7)	1 (0.7)	0.025	0.155
**Hypertension**	79 (28.1)	53 (18.9)	4 (2.9)	1 (0.7)	<0.001	<0.001
**Weight decreased**	78 (27.8)	7 (2.5)	17 (12.2)	0	<0.001	0.061
**Alopecia**	72 (25.6)	0	30 (21.6)	0	0.364	-
**Nausea**	70 (24.9)	1 (0.4)	30 (21.6)	1 (0.7)	0.451	0.611
**Vomiting**	65 (23.1)	2 (0.7)	25 (18.0)	1 (0.7)	0.227	0.993
**Decreased appetite**	64 (22.8)	1 (0.4)	24 (17.3)	1 (0.7)	0.192	0.611
**Constipation**	62 (22.1)	0	25 (18.0)	1 (0.7)	0.332	-
**Urinary tract infection**	62 (22.1)	5 (1.8)	18 (12.9)	0	0.025	0.114
**Hypertriglyceridemia**	58 (20.6)	21 (7.5)	12 (8.6)	1 (0.7)	0.002	0.004
**Diarrhea**	53 (18.9)	3 (1.1)	18 (12.9)	3 (2.2)	0.128	0.375
**COVID-19**	51 (18.1)	0	14 (10.1)	1 (0.7)	0.031	0.319
**Hypoalbuminemia**	50 (17.8)	0	18 (12.9)	0	0.205	-
**Hypercholesterolemia**	49 (17.4)	3 (1.1)	11 (7.9)	0	0.009	0.222
**Pyrexia**	44 (15.7)	0	15 (10.8)	0	0.177	-
**Abdominal pain**	43 (15.3)	2 (0.7)	15(10.8)	0	0.207	0.319
**Lymphocyte count decreased**	42 (14.9)	15 (5.3)	26 (18.7)	7 (5.0)	0.325	0.896
**Epistaxis**	41 (14.6)	0	0	0	<0.001	-
**Hypoesthesia**	41 (14.6)	3 (1.1)	21 (15.1)	1 (0.7)	0.888	0.730
**Blood creatinine increased**	38 (13.5)	0	7 (5.0)	0	0.008	-
**Hyperuricemia**	37 (13.2)	0	4 (2.9)	0	<0.00	-
**Hyponatremia**	37 (13.2)	1 (0.4)	13 (9.4)	0	0.256	0.481
**Cough**	32 (11.4)	1 (0.4)	11 (7.9)	0	0.269	0.481
**Stomatitis**	30 (10.7)	9 (3.2)	5 (3.6)	2 (1.4)	0.098	0.287
**Hyperglycemia**	28 (10.0)	0	5 (3.6)	0	0.023	-
**Abdominal distension**	20 (7.1)	2 (0.7)	14 (10.1)	0	0.296	0.319
**Leading to suvemcitug or placebo dose interruption**	232 (82.6)		86 (61.9)		-	-
**Leading to suvemcitug or placebo dose reduction**	26 (9.3)		0		-	-
**Leading to discontinuation of suvemcitug or placebo**	19 (6.8)		3 (2.2)		-	-

Data are numbers (%) and shown for adverse events that occurred in at least 10% of the participants in either arm during the study intervention or up to 28 days after discontinuation of the intervention. The adverse events were graded according to the NCI CTCAE (v.5.0).

*Two-sided chi-square test; no adjustment for multiple comparisons.

AEs of any grade related to suvemcitug or placebo occurred in 267 (95.0%; grade ≥3: 199 participants, 70.8%) participants in the suvemcitug arm and 124 (89.2%; grade ≥3: 69, 49.6%) participants in the placebo arm (Supplementary Table 3). Serious AEs related to suvemcitug or placebo occurred in 63 participants (22.4%) in the suvemcitug arm and 22 participants (15.8%) in the placebo arm (Supplementary Table 4). No suvemcitug-related grade 5 TEAE occurred.

### Participant-reported outcomes

**Extended Data Fig. 3:**
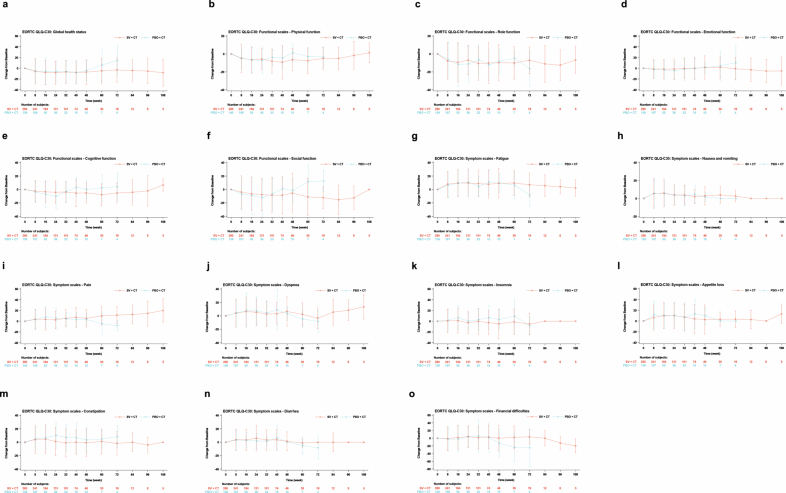
EORTC QLQ-C30 change from baseline over time. **a**, global health scale, functional scales. **b**, physical function. **c**, role function. **d**, emotional function. **e**, cognitive function. **f**, social function and symptom scales. **g**, fatigue. **h**. nausea and vomiting. **i**, pain. **j**, dyspnea. **k**, insomnia. **l**, appetite loss. **m**, constipation. **n**, diarrhea. **o**, financial difficulties. Scores range from 0 to 100; a higher score represents higher (‘better’) global health status/overall quality of life and function or symptoms. EORTC, European Organization for Research and Treatment of Cancer; GHS, global health scale; QLQ-C30, Quality of Life Core 30 questionnaire; QoL, quality of life; SE, standard error; PBO + CT, placebo plus chemotherapy; SV + CT, suvemcitug plus chemotherapy. Source data

**Extended Data Fig. 4:**
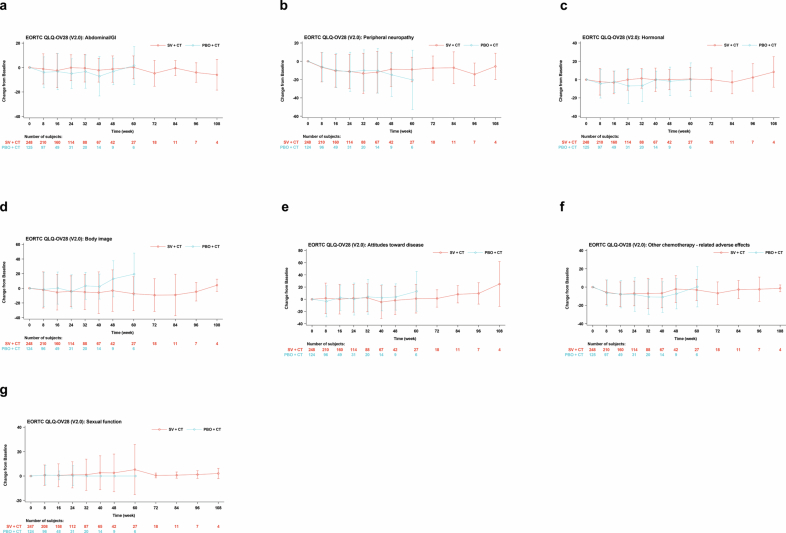
Mean changes in EORTC QLQ Ovarian Cancer Module (EORTC QLQ-OV28) QoL parameters. **a**, QLQ-OV28 abdominal/gastrointestinal symptoms. **b**, peripheral neuropathy. **c**, hormonal. **d**, body image. **e**, attitudes toward disease. **f**, other chemotherapy-related adverse effects. **g**, sexual function. Scores range from 0 to 100; a higher score represents better function or symptoms. EORTC, European Organization for Research and Treatment of Cancer; QoL, quality of life; SE, standard error; PBO + CT, placebo plus chemotherapy; SV + CT, suvemcitug plus chemotherapy. Source data

The European Organization for Research and Treatment of Cancer (EORTC) questionnaires QLQ-OV28 and QLQ-C30 did not differ between the two arms (Extended Data Figs. 3 and 4).

### Exploratory analyses

In total, 11 of 280 participants (3.9%) in the suvemcitug arm with samples at screening were positive for antidrug antibody (ADA) before treatment initiation but none were positive for neutralizing ADA. In total, 38 of 277 participants (13.7%) were positive for treatment-emergent ADA and four (1.4%) were positive for neutralizing ADA. ADAs persisted for ≥16 weeks in 6.1% (17/277) participants.

## Discussion

The SCORES trial met its primary endpoint, demonstrating that suvemcitug, when added to chemotherapy, increased the median PFS from 2.7 to 5.5 months, with a corresponding 54% reduction in the risk of progression or death. At data maturity, a significant OS benefit with suvemcitug was observed, with a 23% reduction in the risk of death.

This trial enrolled a broader population than the AURELIA trial, including participants who previously received bevacizumab and/or a PARP inhibitor^13,21^. In the AURELIA trial^13,21^, 7.2% of the participants received prior antiangiogenic therapy compared to 49.4% in this trial (bevacizumab: 43.7%). Approximately one third (32.3%) of participants in this trial received ≥3 prior lines of systemic therapies, whereas the AURELIA trial excluded persons who received >2 prior lines of systemic therapies. These discrepancies may explain the shorter median PFS (2.7 months) in the placebo arm in this trial. The improvement in PFS by suvemcitug was also supported by the higher response rate and the longer DOR (8.8 months versus 6.1 months in the placebo arm).

Suvemcitug conferred broad PFS benefit across all subgroups in this trial, including participants with previous exposure to PARP inhibitors and/or antiangiogenic therapy. PARP inhibitors have become the standard of care for women with advanced OC, particularly for newly diagnosed persons with a *BRCA* mutation or with homologous recombination deficiency (HRD)-positive tumors^5,22,23^. The efficacy of antiangiogenic therapy for persons with platinum-resistant, recurrent OC who have been previously treated with a PARP inhibitor remains unclear. In this trial, approximately half of the participants (49.4%, 208/421) received prior PARP inhibitor therapy and the proportions of participants who previously received PARP inhibitors were well balanced in the two arms. In subgroup analyses, suvemcitug conferred a significant PFS benefit regardless of previous exposure to PARP inhibitors. Most notably, suvemcitug led to a 51% reduction in the risk of progression or death compared to placebo in participants with previous exposure to PARP inhibitor (HR: 0.49, 95% CI: 0.35–0.69) and a statistically nonsignificant trend of lower risk of death (HR: 0.82, 95% CI: 0.58–1.16), suggesting that suvemcitug could be offered as an effective treatment option in persons with platinum-resistant, recurrent OC who were previously exposed to PARP inhibitors. These findings are consistent with the association between longer PFS and bevacizumab plus chemotherapy (8.9 months versus 3.1 months alone, *P* = 0.022) in a retrospective study in persons with OC who received prior PARP inhibitor^24^ and support the incorporation of suvemcitug into the therapeutic regimens for platinum-resistant recurrent OC, including those were previously treated with a PARP inhibitor.

The efficacy of antiangiogenic therapy in persons with platinum-resistant OC was established in the AURELIA trial of bevacizumab and in subsequent studies, including the TRIAS trial of sorafenib^13,25^, the APPROVE trial of apatinib^26^, a phase 3 trial of pazopanib^27^ and a phase 2 study of anlotinib^28^. The phase 2 APPROVE trial demonstrated a significant improvement in PFS with apatinib combined with liposomal doxorubicin when compared to liposomal doxorubicin only^26^. In the TRIAS study^25^, sorafenib showed a statistically significant and clinically meaningful improvement in PFS in persons with platinum-resistant OC when given orally in combination with topotecan and continued as maintenance therapy. With the increasing use of bevacizumab, however, there is a rising population of persons who have failed prior bevacizumab or anti-VEGF tyrosine kinase inhibitors. There is also a subset of persons who could not tolerate bevacizumab toxicities. In this trial, 43.7% and 49.4% of the participants received prior bevacizumab and antiangiogenic therapy, respectively. In subgroup analysis, suvemcitug demonstrated a clear PFS benefit in participants who were previously treated with an antiangiogenic agent, suggesting that suvemcitug could offer an effective treatment option in persons who have failed antiangiogenic therapy.

Optimal chemotherapy regimen for platinum-resistant, recurrent OC remains an area of uncertainty. In this trial, participants received investigator’s choice of chemotherapy (paclitaxel versus PEGylated liposomal doxorubicin versus topotecan) and randomization was stratified on the basis of chemotherapeutic agent. In all three chemotherapy subgroups, adding suvemcitug to chemotherapy significantly prolonged PFS. In the paclitaxel subgroup, suvemcitug led to a 55% reduction in the risk of progression or death (HR: 0.45, 95% CI: 0.31–0.65). In the PEGylated liposomal doxorubicin subgroup, there was a statistically nonsignificant trend for reduced risk of progression or death (HR: 0.69, 95% CI: 0.45–1.05). Participants receiving paclitaxel appeared to stay on treatment longer than those receiving liposomal doxorubicin (median exposure duration: 22.2 weeks versus 17.3 weeks; Supplementary Table 2). Similarly, in the AURELIA trial, PFS benefit with bevacizumab over placebo was greater in the paclitaxel cohort (HR: 0.46, 95% CI: 0.30–0.71) than the liposomal doxorubicin cohort (5.4 versus 3.5 months; HR: 0.57, 95% CI: 0.39–0.83)^29^. The findings from the AURELIA trial and the current trial, as well as real-world evidence, indicate that the combination of antiangiogenic therapy with paclitaxel is optimal^30^. Experiences with OC and breast cancer indicate that the pairing of bevacizumab and weekly paclitaxel may enhance antiangiogenic activities, resulting in a more pronounced antitumor effect than other chemotherapies^29,31,32^. Given the differential toxicity profile and cost of paclitaxel, topotecan and liposomal doxorubicin, choice of chemotherapy is worthy of further scrutiny. The small number of participants receiving topotecan in this trial makes it difficult to draw firm conclusions.

PFS benefit with suvemcitug was observed in the subgroup of participants with ascites at baseline. VEGF is involved in ascites formation in persons with OC and VEGF inhibition with bevacizumab resulted in improvement in PFS in the AURELIA trial^13,33,34^.^.^ A PFS benefit with suvemcitug was also seen in the subgroups of participants with at least three lines of prior antitumor therapies and with <3-month platinum-free interval in this trial. Considering the fact that these subgroups of participants have very limited therapeutic options, these findings are particularly encouraging.

A key issue in drug development for platinum-resistant OC has been the extent to which PFS benefit translates to an OS benefit^35,36^. Response patterns of targeted therapy including immune therapy and antiangiogenic therapy can differ greatly from traditional anticancer drugs such as chemotherapeutic drugs, with distinct kinetics of survival curves^37,38^. The OS benefit with suvemcitug in this trial was statistically significant albeit modest. The Kaplan–Meier OS curves of the two arms were relatively close during the first half of the trial period but the difference became more apparent as the follow-up time extended, especially after 18 months, supporting long-term survival benefits. Subsequent antitumor therapy may have attenuated the observed advantage in OS, which may have accounted for the modest prolongation of OS over the control arm. A preplanned supplementary analysis of OS in this trial that addressed subsequent antitumor therapy demonstrated that suvemcitug led to a 10.4-month extension of median OS, with a 41% reduction in the risk of death. The findings suggest that suvemcitug, when added to chemotherapy, conferred substantial benefits in terms of both PFS and OS. The robustness of these findings is supported by sensitivity and supplementary analyses, with the use of unstratified and stratified Cox proportional-hazards models.

The safety profile of suvemcitug in this trial is consistent with that reported by early-stage clinical trials^19,20^, with no new safety concerns. The suvemcitug arm had higher rates of any grade TEAEs including neutropenia and thrombocytopenia but grade ≥3 TEAEs did not differ between the two arms with the exception of proteinuria and hypertension, two AEs consistently reported for antiangiogenic agents. The higher rates of TEAEs could be partially attributed to the myelosuppressive effects of longer chemotherapy exposure in the suvemcitug arm than the control arm in our view.

Suvemcitug is a humanized rabbit monoclonal IgG1 (*κ*) antibody and, except for the complementarity-determining region, has a similar sequence to bevacizumab. During the trial, ADA against suvemcitug was measured during treatment and up to 28 days after the last dose. ADA was identified in 13.7% of the participants in the suvemcitug arm but the rate of neutralizing ADA was low (1.4%).

This trial differs from the AURELIA trial in two key aspects. First, the AURELIA trial had an open-label design and the primary endpoint of PFS was assessed by investigators; in contrast, the current trial was double-blinded, with the primary endpoint of PFS evaluated by the BIRC. Second, participants in the AURELIA trial had no more than two prior lines of systemic treatment, none received prior PARP inhibitor therapy and only 7.2% of the participants were previously treated with bevacizumab; in contrast, participants in the current trial had up to six prior lines of systemic treatment and nearly half of the participants were previously treated with a PARP inhibitor and an antiangiogenic agent.

This trial had several limitations. Firstly, the exclusive recruitment of participants within China may limit the generalizability of findings to broader global populations. Secondly, this trial excluded persons who received ≥2 lines of systemic therapy for OC after platinum resistance, as well as persons with primary platinum-refractory OC who progressed during the first platinum-based chemotherapy, limiting applicability to the most heavily pretreated or more aggressive disease states. Thirdly, the trial did not did not examine germline and somatic *BRCA* mutations (or other HRD-related factors). Lastly, the COVID-19 pandemic caused notable disruptions in study treatment and assessment.

In conclusion, the addition of suvemcitug to chemotherapy led to a significant improvement in both PFS and OS in persons with platinum-resistant OC, with a manageable safety profile and no unexpected toxicities. The findings suggest that suvemcitug should be incorporated as a part of standard treatment in persons with platinum-resistant OC, including those who have received bevacizumab/PARP inhibitor.

## Methods

The trial protocol and amendments were approved by the ethics committees of all participating centers (master protocol approved by the Ethics Committee of Cancer Hospital, Chinese Academy of Medical Sciences and Peking Union Medical College). A full list of participating centers are available at ClinicalTrials.gov (NCT04908787). All participants provided written informed consent before any trial-related activities. The trial was conducted according to the Declaration of Helsinki and the Good Clinical Practice guidelines.

### Study design and participants

This randomized, double-blind, placebo-controlled, phase 3 trial (SCORES) was conducted at 55 tertiary-care centers in China. Recruitment was conducted by screening persons seeking medical attention during daily practice. The inclusion criteria were as follows:

1. Age ≥ 18 years;
2. Histologically confirmed epithelial OC, fallopian tube cancer or primary peritoneal cancer; pathological types were high-grade serous adenocarcinoma, endometrioid carcinoma (G2 or G3), mixed epithelial carcinoma (high-grade serous adenocarcinoma and G2/G3 endometrioid carcinoma components had to account for more than 50%), malignant Brunner’s tumor, undifferentiated carcinoma, dedifferentiated carcinoma and other rare types such as mesonephric duct-like carcinoma, gastric adenocarcinoma;
3. Persons with platinum-resistant recurrent OC who received a platinum-containing regimen and progressed on a platinum-containing regimen (platinum-refractory) or had a time to relapse of <6 months (184 calendar days) from the end of platinum-containing therapy (at least four cycles) until 28 days after the last dose.
  Definition of relapse or progression (any of the following):
  - Documented radiographic progression;
  - Persistent elevation of cancer antigen 125 (CA-125 ≥ 2 times upper limit of normal (ULN) and confirmed 1 week later) with clinical symptoms or physical examination suggestive of disease progression;
4. Progression during or after the most recent line of systemic therapy or intolerable therapy and at least one measurable lesion (assessed by investigator according to RECIST v.1.1) within 4 weeks before randomization;
5. ECOG performance score of 0–1 within 7 days before the first dose;
6. Previous chemotherapy ended ≥3 weeks from the first dose of this study, monoclonal antibody antitumor therapy ended ≥4 weeks from the first dose of this study, and small-molecule targeted therapy ended ≥2 weeks from the first dose of this study;
7. Treatment-related AEs recovered to the National Cancer Institute (NCI) Common Terminology Criteria for Adverse Events (CTCAE) grade ≤1 (except grade 2 alopecia);
8. Adequate organ function and meeting all of the following laboratory test results before enrollment:
  - Bone marrow (no blood transfusion or blood products, granulocyte colony-stimulating factor or other hematopoietic stimulating factors were not used for correction within 14 days before blood routine examination during the screening period): neutrophils ≥ 1.5 × 10^9^ L^−1^, hemoglobin ≥ 90 g L^−1^, platelets ≥ 100 × 10^9^ L^−1^;
  - Liver function: total bilirubin ≤ 1.5 × ULN, aspartate aminotransferase ≤ 3 × ULN, alanine aminotransferase ≤ 3 × ULN, alkaline phosphatase ≤ 3 × ULN; if liver metastasis, aspartate aminotransferase ≤ 5 × ULN, alanine aminotransferase ≤ 5 × ULN;
  - Renal function: serum creatinine ≤ 1.5 ULN or creatinine clearance ≥ 60 ml min^−1^ calculated according to the Cockroft–Gault formula;
  - Coagulation: international normalized ratio (INR) ≤ 1.5 (INR range should be 2–3 if individual is on a stable dose of warfarin for venous thrombosis management), activated partial thromboplastin time ≤ 1.5 ULN;
9. Estimated survival time ≥ 12 weeks;
10. Women of childbearing age agreed to remain abstinent or used contraception with an annual failure rate of <1% during treatment and for at least 6 months following the last dose of suvemcitug, placebo, paclitaxel, liposomal doxorubicin or topotecan, whichever occurred later.

The exclusion criteria were as follows:

1. Received >1 lines of systemic therapy for OC after platinum resistance and/or >1 lines of nonplatinum systemic therapy before platinum resistance;
2. Progression during the first platinum-based chemotherapy (from first dose to within 28 days after last dose);
3. Ovarian epithelial tumors with low malignant potential, such as low-grade serous adenocarcinoma, borderline tumors;
4. Ovarian mucinous carcinoma or clear cell carcinoma;
5. Nonepithelial tumors, such as sex cord and stromal tumors, germ cell tumors, carcinosarcoma;
6. Persons with other active malignant tumors within 5 years or at the same time (cured localized tumors, such as cutaneous basal cell carcinoma, cutaneous squamous cell carcinoma or cervical carcinoma in situ, can be enrolled);
7. Any pelvic or abdominal radiotherapy;
8. Recent major surgery or anticipated surgical intervention:
  A) Major surgery or notable trauma within 28 days before enrollment;
  B) Major surgical procedures anticipated during the course of the study, including but not limited to abdominal surgery (laparotomy or laparoscopy) before disease progression;
  C) Open biopsy performed within 7 days before enrollment;
9. Known hereditary or acquired bleeding and thrombophilia (for example, hemophilia, coagulopathy, thrombocytopenia or hypersplenism); clinically notable bleeding events, arterial or deep venous thromboembolic events or superficial venous thrombosis and myenteric venous thrombosis requiring intervention within 6 months before enrollment;
10. Taking aspirin (>325 mg per day) currently or recently (within 10 days before first dose);
11. Persons with a history of intestinal obstruction (including incomplete intestinal obstruction) within 3 months before enrollment; persons with a history of abdominal fistula, gastrointestinal perforation or abdominal abscess; persons with intestinal invasion found by imaging examination (computed tomography, magnetic resonance imaging) or pelvic examination during the screening period;
12. Severe infection requiring systemic antibiotic infusion or hospitalization during the screening period;
13. Persons with clinically manifested central nervous system disease, brain metastasis, stroke or transient ischemic attack within 6 months before enrollment;
14. Clinically notable cardiovascular disease:
  - Uncontrolled hypertension (defined as systolic blood pressure ≥ 150 mmHg and/or diastolic blood pressure ≥ 100 mmHg after drug treatment);
  - History of myocardial infarction or unstable angina within 6 months before enrollment;
  - New York Heart Association class II and above heart failure;
  - Severe arrhythmia requiring medication, excluding asymptomatic atrial fibrillation with controlled ventricular rate;
15. Left-ventricular ejection fraction < 50%;
16. Presence of neuropathy grade ≥2 (CTCAE 5.0) at screening;
17. Presence of severe nonhealing wound, ulcer or fracture; serous effusion (including pleural effusion and pericardial effusion) with clinical symptoms and requiring surgical treatment; difficult-to-control ascites;
18. Known serious hypersensitivity to the therapeutic agents or excipients used in the trial;
19. Pregnant or lactating women;
20. Persons with proteinuria (urine protein > 1 found in screening examination or urine protein > 1 that failed to return to normal within 24 h after retest);
21. Currently participating in another clinical study or planning to start treatment in this study less than 30 days before the end of treatment in the previous clinical study;
22. Other conditions that the investigator considered inappropriate for participation in this study.
23. Persons who have previously used BD0801. Participants were required to have platinum-refractory or resistant disease. Other key inclusion criteria included ≥1 measurable lesions according to the investigators per RECIST (v.1.1), an ECOG performance status of 0–1 and adequate hematologic and organ function. Persons who had primary platinum-refractory disease or who received ≥2 lines of systemic therapy for OC after platinum resistance were excluded. The full eligibility criteria are available in the trial protocol. Trial reporting followed the CONSORT 2010 statement^39^.

### Randomization and masking

Participants were randomized (2:1) to receive the investigator’s choice of chemotherapy plus suvemcitug or placebo using a minimization technique. Randomization was conducted using an interactive web response system and stratified according to platinum-refractory status (yes versus no), number of prior systemic therapies (one versus two), chemotherapeutic agent (paclitaxel versus PEGylated liposomal doxorubicin versus topotecan) and prior antiangiogenic therapy (yes versus no). Participants were enrolled and assigned to interventions by site investigators. Investigators, participants and the sponsor were blinded to allocation assignment.

### Procedures

Suvemcitug (1.5 mg kg^−1^) or placebo was infused on days 1 and 15 of each 4-week cycle. Paclitaxel (80 mg m^−^^2^; days 1, 8, 15 and 22), topotecan (4 mg m^−^^2^; days 1, 8 and 15) or PEGylated liposomal doxorubicin (40 mg m^−^^2^; day 1) was given intravenously every 4 weeks. Treatments were continued until disease progression, unacceptable toxicities, withdrawal of consent or death. Participants who ended treatment were followed up every 3 months for data on subsequent antitumor treatment and survival. Dose modifications of suvemcitug and chemotherapeutic drugs were allowed at the discretion of investigators. Two levels of dose modifications were permitted for suvemcitug (1.5 mg kg^−1^ to 1.0 mg kg^−1^ and 1.0 mg kg^−1^ to 0.5 mg kg^−1^). Other protocol-mandated treatment changes are available in the trial protocol.

Tumor response was assessed radiologically by BIRC and investigators per RECIST (v.1.1) at baseline and every 8 weeks for the first 48 weeks and every 12 weeks thereafter until disease progression by BIRC, start of new antitumor therapy, death or withdrawal from the study, whichever occurred first.

Safety was assessed throughout the study using the NCI CTCAE (v.5.0). The occurrences, frequencies and severities of AEs were tabulated and all AEs were described in MedDRA (v.27.1) preferred terms and CTCAE grade.

Quality of life was assessed with the use of the EORTC QLQ-C30 and QLQ-OV28 questionnaires.

### Outcomes

The primary endpoint was BIRC-assessed PFS, defined as the time from randomization to the first radiologically documented tumor progression or death, whichever occurred first, per RECIST (v.1.1). The key secondary endpoint was OS, defined as the interval from randomization to death of any cause. Other secondary endpoints included objective response rate (ORR), disease control rate (DCR), DOR, defined as the time from the first confirmed complete response or partial response to the first documented progressive disease or death of any cause, and quality of life. ORR, DCR and DOR were assessed by investigators and the BIRC. Safety endpoints included the incidence of AEs and serious AEs. Other endpoints including pharmacokinetics of suvemcitug and anti-suvemcitug antibodies will be reported elsewhere.

### Statistics and reproducibility

The planned sample size was 411. The statistical power was based on the total number events of PFS per RECIST (v.1.1) by BIRC and OS. Assuming a treatment effect HR of 0.69, corresponding to an improvement in median PFS from 4.4 months in the placebo arm to 6.4 months in the suvemcitug arm, 304 PFS events would provide 87% power to detect the PFS treatment effect at one-sided significance level of 0.025. For the key secondary endpoint of OS, 278 events would provide 80% power to detect an HR of 0.70, corresponding to an improvement in median OS from 13.3 months in the placebo arm to 19.0 months in the suvemcitug arm. The familywise type I error was controlled in a fixed sequential testing manner, that is, the OS was tested only if the treatment effect of PFS was statistically significant at a one-sided α level of 0.025. An administrative one-sided α level of 0.0001 would be spent on the OS analysis along with PFS primary analysis.

Efficacy was assessed in the full analysis set, which included all randomized participants, when 304 PFS events had occurred in all randomized participants. The second analysis was performed when 278 OS events occurred. PFS and other time-to-event endpoints were analyzed using the Kaplan–Meier method and the corresponding 95% CIs for median time were calculated using the Brookmeyer–Crowley method. The primary hypothesis for BIRC-assessed PFS was evaluated using a stratified log-rank test. HRs were estimated using a stratified Cox proportional-hazards model with Efron’s method for tie handling. Unstratified HRs were calculated as well. ORR and DCR were estimated for each arm, along with their two-sided 95% CIs, using the Clopper–Pearson method. The rate differences between arms were calculated using the Miettinen–Nurminen methods^40^. Prespecified subgroup analyses of PFS by BIRC and OS were conducted using similar methods to those for the primary endpoint. Sensitivity and supplementary analyses for PFS were performed as specified in the statistical analysis plan. At the final analysis, the actual values of corrected stratification factors were used for stratified analyses. Given the use of dynamic randomization method without increasing type I error, log-rank test *P* values were calculated using the rerandomization method^41^ and original log-rank test *P* values served as nominal *P* values. Supplementary analyses for OS were carried out using a hypothetical strategy by censoring subsequent antitumor therapy.

The Cox regression is based on the proportional-hazards model assumption. Before analysis, the proportional-hazards assumption was verified for the following endpoints in this study: BIRC-assessed PFS and investigator-assessed PFS and OS.

The safety set included participants who received at least one dose of the study medications. An independent data monitoring committee monitored the ongoing safety data until the first analysis.

All analyses and data processing were completed using SAS (v.9.4).

### Reporting summary

Further information on research design is available in the Nature Portfolio Reporting Summary linked to this article.

## Supplementary information

Supplementary Information Trial protocol and statistical analysis plan.

Reporting Summary

Supplementary Tables 1–4 Supplementary Tables 1–4.

## Source data

Source Data Fig. 2 Survival outcomes, as assessed by an independent review committee.

Source Data Fig. 3 Forest plot showing the results of subgroup analyses.

Source Data Fig. 4 Waterfall plot showing treatment responses.

Source Data Extended Data Fig. 1 Subgroup analyses of PFS based on prior exposure to PARP inhibitors.

Source Data Extended Data Fig. 2 Supplementary analyses of OS.

Source Data Extended Data Fig. 2 Subgroup analyses of OS.

Source Data Extended Data Fig. 3 EORTC QLQ-C30 change from baseline over time.

Source Data Extended Data Fig. 4 Mean changes in EORTC QLQ OC module (EORTC QLQ-OV28) quality-of-life parameters.

## Data Availability

The trial protocol and statistical analysis plan are available in the Supplementary Information. All other data supporting the findings of this study (detailed AEs and Kaplan–Meier curves in the subgroup analyses) are available from the corresponding authors on reasonable request. Source data are provided with this paper.

## Code availability

There was no custom code or mathematical algorithm central to this study’s conclusion.
