# Supplementary material for: Suvemcitug plus chemotherapy in women with platinum-resistant recurrent ovarian cancer: the SCORES randomized, double-blinded, phase 3 trial
Source: Nat Cancer. 2026 Jan 9;7(1):182–93. doi: 10.1038/s43018-025-01085-z (PMC12858396; doi:10.1038/s43018-025-01085-z)
Supplement: Supplementary file 1 — Trial protocol and statistical analysis plan. [file 43018_2025_1085_MOESM1_ESM.pdf]

nature cancer

Supplementary information https://doi.org/10.1038/s43018-025-01085-z

Suvemcitug plus chemotherapy in women with platinum-resistant recurrent ovarian cancer: the SCORES randomized, double-blinded, phase 3 trial

In the format provided by the authors and unedited

as non-target lesions. Pathological status < 10 mm was not recorded.

(2) Criteria for Response

Evaluation of target lesions

CR: disappearance of all target lesions. All lymph nodes must be non-pathological (< 10 mm short axis).

PR: > 30% decrease in sum of lesion lengths, taking baseline as control.

PD: > 20% increase in sum of long lesions and an absolute increase of at least 5 mm, taking as reference the smallest sum; or the appearance of one or more new lesions.

SD: Decrease but not PR or increase but not PD in the sum of long diameters of baseline lesions, taking as reference the smallest sum diameters.

Evaluation of non-target lesions

CR: disappearance of all non-target lesions and normal levels of tumor markers. All lymph nodes must be non-pathological (< 10 mm short axis).

Non-CR/Non-PD: Persistence of one or more non-target lesions and/or tumor markers above normal.

PD: Appearance of one or more new lesions or/and progression of non-target lesions.

(3) New lesions

The finding of new lesions must be very clear. The lesions found at follow-up that were not present at baseline were new lesions. If a new lesion is identified that requires repeat testing for confirmation because it is small, the time to report PD after confirmation should be the time of initial discovery.

3. Overall efficacy evaluation

(1) Efficacy Assessments

Overall response evaluation can be obtained by combining target lesion evaluation with non-target lesion evaluation (see the table below). Best overall response is the best response recorded from the start of treatment until disease progression or relapse. Generally, the patient 's best response evaluation relies on criteria for measurement and confirmation.

Target lesions	Non-target lesions	New Lesion	Overall evaluation of

			efficacy
CR	CR	None	CR
CR	Non-CR/Non-PD	None	PR
CR	Not evaluable	None	PR
PR	Non-PD or Not all evaluated	None	PR
SD	Non-PD or Not all evaluated	None	SD
Not all evaluated	Non-PD	None	NE
PD	Any	Yes or No	PD
Any	PD	Yes or No	PD
Any	Any	Yes	PD

Non-target lesions	New Lesion	Overall evaluation of efficacy
CR	None	CR
Non-CR/Non-PD	None	Non-CR/non-PD
Not all evaluated	None	NE
Definite PD	Yes or No	PD
Any	Yes	PD

(2) Frequency of tumor re-evaluation

The frequency of tumor re-evaluation depends on the treatment regimen, and in fact the time to benefit of treatment is unclear, and re-evaluation every 2 cycles (6 to 8 weeks) is reasonable and should be adjusted to shorter or longer times in special cases. After the end of treatment, whether the tumor needs to be re-evaluated depends on whether the endpoint of the clinical trial is response rate or time to event (TTE), such as TTP and PFS. In the case of the latter, routine repeated assessments are required, and the interval between secondary assessments is not strictly specified.

(3) Confirmation of efficacy

In non-randomized trials with response rate as the primary endpoint, patients with PR and CR should be rechecked and confirmed 4 weeks after the first evaluation. SD patients must meet the criteria for lesion stabilization at least once after study entry as measured during follow-up, and the minimum time interval for duration of stable disease is usually

no less than 6 weeks.

(4) Disease in remission

It is measured from the time of the first measurement of CR or PR until the first recurrence or progression of disease.

(5) Stable disease

Time from start of treatment to disease progression.

Response period, stable period and PFS are affected by the follow-up frequency after baseline evaluation. Because they are affected by multiple factors such as disease type, stage, treatment cycle and clinical practice, the basic follow-up frequency cannot be determined so far, which affects the accuracy of trial endpoint indicators to some extent.

(6) PFS

In some cases, response rate may not necessarily be the best method to evaluate response, and PFS may be an ideal surrogate. However, phase II trials using PFS as an endpoint are best randomized controlled trials. Increasing numbers of phase III clinical trials in progressive tumors have used PFS/TTP as the primary endpoint. However, trials often have to limit the number of patients enrolled to have measurable disease. If a large proportion of patients are excluded, whether the results are representative will become a problem, and the speed of enrollment will also have a great impact. Some trials do not exclude patients without measurable disease, but care should be taken to determine disease progression in such patients.

(7) Independent Committee of Experts

For objective response rates where CR/PR is the primary endpoint and there are not many cases, it is recommended that all responses must be checked by an independent expert committee outside of the study. In case of randomized trials, the independent reviewer will be blinded. It is best to review the patient 's data and imaging data at the same time.

4. Result Reporting

(1) Phase II clinical trial

If response rate is the primary endpoint, measurable disease must be present in all enrolled patients, and results must be reported for all patients, even if there is a deviation

from the primary study protocol or the case is not evaluable. Each patient was to be reported as CR, PR, SD, PD, or NE (not evaluable with reasons). Generally, all eligible patients are used as denominators for calculation of response rates (sometimes all treated patients are used as denominators), and evaluable cases should not be used as denominators.

(2) Phase III clinical trial

Evaluation of response rate in Phase III clinical trials can be used as a measure of relative antitumor activity and almost always as a secondary endpoint because the observed difference in response rates does not necessarily generalize to clinical benefit. Response rate will only be considered as the primary endpoint if objective response rate is directly related to clinical benefit, but all enrolled patients must have measurable disease.

When response rate is a secondary endpoint and not all patients have measurable disease, the overall best response rate must be pre-specified in the protocol. Response rates may be reported for the ITT dataset (all randomized patients) or for the set of patients with measurable disease at baseline, but must be pre-specified in the protocol.

Statistical Analysis Plan, SAP

**Protocol Name:**	**A Randomized Double-Blind Phase III Study of BD0801 (Suvemcitug) for Injection in Combination With Chemotherapy Versus Placebo in Combination With Chemotherapy in Patients With Recurrent Epithelial Ovarian, Fallopian Tube, and Primary Peritoneal Cancer Who Have Failed Platinum-based Chemotherapy**
**Protocol No.:**	**SIM-63-OC-301**
**Study Phase:**	**Phase III**
**Sponsor:**	**Shanghai Xianxiang Medical Technology Co., Ltd.**
**SAP Author:**	**Haolin Sun**
**SAP Version:**	**2.0**
**SAP Version Date:**	**29DEC2023**

Confidentiality Statement

This protocol and all relevant information involved in it are confidential and proprietary property of Shanghai Xianxiang Medical Technology Co., Ltd. Receiving this document means that the recipient agrees that no unpublished information contained herein may be published or disclosed without the written consent of Shanghai Xianxiang Pharmaceutical Technology Co., Ltd., unless such disclosure is required by applicable laws and regulations.

Revision History

Version No.	Date Formulated (Version Date)	Revised Content
V1.0	2022-08-03	NA
V2.0	2023-12-29	Corresponding amendment according to protocol update:<br>1. Protocol 5.1 version changed the name of the sponsor from "Jiangsu Simcere Pharmaceutical Co., Ltd." to "Shanghai Xianxiang Medical Technology Co., Ltd.".<br>2. Protocol 5.0 was adapted to the ESTIMAND framework according to ICH E9R1 requirements, and the structure was adjusted accordingly in SAP to detail the main estimation target and key secondary estimation targets.<br>3. Protocol 5.0 version set OS as a key secondary endpoint, added sample size calculation rationale for OS, and added multiplicity control. Added time points for analysis, ie, primary analysis for PFS and primary analysis for OS. In order to ensure the integrity of the trial, Protocol 5.0 added an internal sponsor unblinded team to conduct the PFS primary analysis.<br>4. The sample size was adjusted from 357 to 411 in Protocol Version 5.0 accordingly. Protocol Version 5.2 considered actual screening and other reasons, and finally may enroll no more patients than 10% of the estimated total sample size.<br>5. The definition of FAS was updated in Protocol Version 4.0 and subjects who received study treatment or not were included in the FAS analysis set according to the ITT principle. The SS set was more specific, and patients who received at least one dose of study treatment (suvemcitug or placebo or paclitaxel, liposomal doxorubicin, topotecan hydrochloride) were included in the SS analysis set. The adjusted EEAS set in protocol version 5.0 includes subjects who died due to disease progression prior to the first disease assessment.<br>6. Protocol 5.2 version added analysis of liver injury and analysis of

Version No.	Date Formulated (Version Date)	Revised Content
		Hy 's law.<br>Revised based on blinded data review:<br>7. Further refine the definition of baseline, imputation rules for missing dates, and range of baseline disease characteristics.<br>8. The relative dose intensity calculation method for dosing, including duration of dosing, was further refined.<br>9. Given the large number of stratification factors and strata, some strata will be considered combined or removed when the model fails to converge.<br>10. Additional analysis 4 for OS, changed from the original primary strategy to subgroup analysis (whether anti-vascular therapy was used in the posterior line).<br>11. The calculation method for the rate difference between groups will be specified and will be performed using Miettinen and Nurminen method with Cochran-Mantel-Haenszel weights.<br>12. Add DOR censoring rules<br>13. Refine the analysis of quality of life endpoints, clarify the subject range based on completion rate, and include the visit range for analysis<br>14. Adverse event summaries will not include adverse events with a preferred term of "disease progression", but adverse events with a preferred term of "disease progression" will be tabulated<br>15. Refined immunogenicity assays and added definition and analytical methods for TEADA.<br>16. Added analysis of treatment-emergent adverse events occurring during the new coronavirus epidemic.

STATISTICAL ANALYSIS PLAN SIGNATURE FORM

Protocol No.	SIM-63-OC-301
Protocol title	A Randomized Double-Blind Phase III Study of BD0801 (Suvemcitug) for Injection Combined with Chemotherapy versus Placebo Combined with Chemotherapy in Patients with Recurrent Epithelial Ovarian, Fallopian Tube, and Primary Peritoneal Cancer Who Have Failed Platinum-based Chemotherapy
DOCUMENT TYPE	☐Statistical Analysis Plan<br>☒Statistical Analysis Plan Amendment
Document Version	V2.0
Author of Statistical Analysis Plan	Haolin Sun
Author Title	Associate Biostatistician Director
Author Signature	
Statistical Analysis Plan Approved By	Sun Shuguang
Approved By Title	Senior Director, Clinical Statistics and Data Management
APPROVED BY	
Approved Date	

CONTENTS

STATISTICAL ANALYSIS PLAN SIGNATURE FORM 4
CONTENTS 5
1. Introduction 10
2. TEST SUMMARY 10
2.1. Study objectives and endpoints 10
2.2. Master Estimate Target .... 错误！未定义书签。
2.2.1. Scientific issues 11
2.2.2. Primary Estimated Target Attribute 11
2.3. Key Secondary Estimated Objectives 12
2.3.1. Scientific issues 12
2.3.2. Critical Secondary Estimated Target Attribute 12
2.4. Study Design 12
2.4.1. Study Plan and Design 13
2.4.2. Randomization and Blinding 14
3. Sample Size Calculation 15
4. Analysis Set 17
4.1. Efficacy Analysis Set 17
4.2. Safety Analysis Set 17
4.3. Efficacy Evaluable Analysis Set 17
4.4. Pharmacokinetic Analysis Set 17
4.5. Immunogenicity Analysis Set 18
5. Statistical Analysis Methods 18
5.1. General analytical provisions 18
5.1.1. Presentation of Results 18
5.1.2. Data processing 19
5.2. Study Population Characteristics 22
5.2.1. Subject disposition 22

5.2.2. Protocol Deviations ... 22

5.2.3. Demographic and Baseline Disease Characteristics ... 22

5.2.4. Past Medical History, Prior Medications, and Prior (Non-Drug) Therapies ... 23

5.2.5. Drug exposure and compliance ... 24

5.2.6. Concomitant Medications and Concomitant Non-Drug Therapies . 25

5.2.7. Subsequent antineoplastic therapy ... 25

5.3. Efficacy analysis ... 25

5.3.1. Primary Efficacy Endpoint ... 26

5.3.2. Key Secondary Efficacy Endpoints ... 30

5.3.3. PFS per RECIST 1.1 by Investigator ... 32

5.3.4. Investigator-assessed ORR and DCR per RECIST 1.1 ... 32

5.3.5. Investigator-assessed DCR per RECIST 1.1 ... 33

5.3.6. OR R per RECIST 1.1 by BIRC ... 33

5.3.7. DCR per RECIST 1.1 by BIRC ... 33

5.3.8. Investigator-assessed DOR per RECIST 1.1 ... 33

5.3.9. DOR per RECIST 1.1 by BIRC ... 34

5.3.10. Quality of Life Endpoints ... 34

5.3.11. Multiplicity correction ... 35

5.4. Safety analysis ... 35

5.4.1. Adverse Events ... 36

5.4.2. Laboratory tests ... 38

5.4.3. Vital signs ... 40

5.4.4. ECOG score ... 40

5.4.5. Physical examination ... 40

5.4.6. ECG examination ... 40

5.4.7. Echocardiography ... 41

5.4.8. Death ... 41

5.5. Analysis Time Point 41
5.6. Pharmacokinetic (PK) Analysis 41
5.6.1. Pharmacokinetic Data Processing 42
5.6.2. Serum drug concentration analysis 43
5.7. Immunogenicity Analysis 43
5.8. Exploratory Analyses 44
5.9. Other analyses 45
5.10. Data Monitoring Committee 46
6. Change from Protocol 46
7. References 48
8. APPENDICES 49
Appendix 1 Protocol Synopsis 49
Appendix 2 TEST FLOW CHART 60
Appendix 3 PFS Censoring Rules 66
Appendix 4 Reason for Censoring Category 68
Appendix 5 Last Known Survival Date 69
Appendix 6 Adverse events of special interest 70

Abbreviations

Abbreviation	Full Name in Chinese

Abbreviation	Full Name in Chinese
**APTT**	Partial prothrombin time
**ADA**	Anti-drug antibody
**AE**	Adverse Events
**AESI**	Adverse events of special interest
**ALB**	Albumin
**ALP**	Alkaline phosphatase
**ALT**	Alanine aminotransferase
**AST**	Glutamic oxaloacetic transaminase
**AUC**	Area under curve
**BIRC**	Blinded Independent Imaging Review Committee
**BLQ**	Below lower limit of quantitation
**BOR**	Overall best response
**BUN**	Urea nitrogen
**Ca**	Calcium
**Cl**	Chlorine
**Cr**	Creatinine
**CrCl**	Creatinine clearance
**CRF**	Case Report Form
**CTCAE**	Criteria for Common Adverse Events
**CV%**	Arithmetic coefficient of variation
**DBIL**	Direct bilirubin
**DCR**	Disease control rate
**ECG**	Electrocardiogram
**ECOG**	Eastern Cooperative Oncology Group
**EDC**	Electronic data capture system
**EEAS**	Efficacy Evaluable Analysis Set
**EGFR**	Glomerular filtration rate
**ESTIMAND**	Estimated target
**FAS**	Full Analysis Set
**GLU**	Glucose
**Hb**	Hemoglobin
**HCT**	Hematocrit
**HR**	Risk ratio
**IAS**	Immunogenicity Analysis Set
**IDMC**	Independent Data Monitoring Committee
**IEC**	Ethics Review Committee
**INR**	International normalized ratio
**ITT**	Intent to Treat
**MedDRA**	Medical Dictionary for Regulatory Activities
**Nab**	Neutralizing antibody

Abbreviation	Full Name in Chinese
**NYHA**	New York Heart Association
**ORR**	Objective response rate
**OS**	Overall survival
**PFS**	Progression-free survival
**PKS**	Pharmacokinetic Analysis Set
**PPS**	Per-Protocol Analysis Set
**PRO**	Patient Reported Outcome Measures
**PT**	Preferred Term
**QOL**	Quality of life
**RECIST**	Criteria for Clinical Response Evaluation in Solid Tumors
**RPSFTM**	Rank Keeping Structural Failure Time Model
**SAE**	Serious Adverse Events
**SAP**	Statistical Analysis Plan
**SOC**	System Organ Class
**SS**	Safety Analysis Set
**TEADA**	Treatment-induced anti-drug antibodies
**TEAEs**	Treatment-emergent adverse event
**TESAE**	Treatment-emergent serious adverse events
**TRAE**	Study Treatment-Related Adverse Events
**ULN**	Upper limit of normal
**WHODrug**	World Health Organization Drug Dictionary

1. Introduction

The analyses and corresponding statistical methods planned for the SIM -63 -OC-301 study (Protocol Version No.: V5.2, Version Date: 26July2023) are provided in this Statistical Analysis Plan (SAP). The study drug, Suvatinib (formerly known as Suvemcitug, R&D code: BD0801), is in the regimen of Suvemcitug used in this SAP.

2. TRIAL SUMMARY

2.1. Study objectives and endpoints

The study objectives and study endpoints for this study are presented inTable 2.1.1 .

Table 2.1.1 Study Objectives and Endpoints

Study objectives	Study Endpoints
**Primary study objectives and primary study endpoints**	
To evaluate the efficacy of suvemcitug plus chemotherapy versus placebo plus chemotherapy (paclitaxel, liposomal doxorubicin, or topotecan) in patients with recurrent epithelial ovarian, fallopian tube, and primary peritoneal cancer who have failed platinum-based chemotherapy.	Progression Free Survival (PFS) per RECIST 1.1 by Blinded Independent Imaging Review Committee (BIRC)
**Secondary study objectives and secondary study endpoints**	
1. To evaluate the efficacy of suvemcitug combined with chemotherapy versus placebo combined with chemotherapy (paclitaxel, liposomal doxorubicin, or topotecan) in patients with recurrent epithelial ovarian, fallopian tube, and primary peritoneal cancer who have failed platinum-based chemotherapy.<br>2. To evaluate the safety of suvemcitug combined with chemotherapy versus placebo combined with chemotherapy (paclitaxel, liposomal doxorubicin, or topotecan) in patients with recurrent epithelial ovarian, fallopian tube, and primary peritoneal cancer who have failed platinum-based chemotherapy.<br>3. To evaluate the effect of suvemcitug combined with chemotherapy versus placebo combined with chemotherapy (paclitaxel, liposomal doxorubicin, or topotecan) on the quality of life in patients with recurrent epithelial ovarian cancer, fallopian tube cancer, and primary peritoneal cancer who have	1. overall survival (OS), which is a key secondary endpoint<br>2. PFS assessed by investigator according to RECIST 1.1 criteria<br>3. Objective response rate (ORR) assessed by investigator according to RECIST 1.1 criteria<br>4. ORR per RECIST 1.1 by BIRC<br>5. disease control rate (DCR) assessed by investigator according to RECIST 1.1 criteria<br>6. DCR assessed by BIRC according to RECIST 1.1 criteria<br>7. duration of response (DOR) as assessed by the investigator according to RECIST 1.1 criteria<br>8. DOR assessed by BIRC according to RECIST 1.1 criteria<br>9 . Incidence of adverse events (AEs) and serious adverse events (SAEs)

failed platinum-based chemotherapy.<br>4. To assess the pharmacokinetic (PK) profile and immunogenicity profile of suvemcitug administered in combination with chemotherapy (paclitaxel, liposomal doxorubicin, or topotecan) in patients with recurrent epithelial ovarian cancer, fallopian tube cancer, and primary peritoneal cancer who have failed platinum-based chemotherapy regimens.	10 . Quality of life endpoints: change from baseline in EORTC QLQ-C30 and EORTC QLQ-OV28 functional scale scores (as assessed by the subject) after treatment;<br>11. pharmacokinetic evaluation: pharmacokinetic parameters after subjects receive suvemcitug in combination with chemotherapy (paclitaxel, liposomal doxorubicin, or topotecan)<br>12. Immunogenicity Evaluation: Immunogenicity Response in Subjects Receiving Combination Chemotherapy with Suvemcitug (Paclitaxel, Liposomal Doxorubicin, or Topotecan)
**Exploratory study objectives and exploratory study endpoints**	
To explore the correlation between PK characteristics of suvemcitug and efficacy and safety.	Correlation of PK profiles of suvemcitug with efficacy and safety
To explore changes in CA-125 from baseline values in subjects and correlation with efficacy.	Change from Baseline Values and Correlation with Efficacy for CA-125 in Subjects

2.2. Primary ESTIMAND

2.2.1. Primary Scientific Question of Interest

The main clinical question of this study is the progression-free survival improvement for suvemcitug plus chemotherapy compared to placebo plus chemotherapy, regardless of discontinuation during treatment, whether concomitant medication/therapy is used or not, assuming no new anti-tumor therapy is used.

2.2.2. Primary Estimand

The primary Estimand attributes are shown in Table 2.2.1.

Table 2.2.1 Primary Estimand Attributes

Attribute	DESCRIPTION
Population	Patients with recurrent epithelial ovarian cancer, fallopian tube cancer, and primary peritoneal cancer who met the inclusion and exclusion criteria and failed platinum-based chemotherapy
Treatment	Suvemcitug plus chemotherapy vs placebo plus chemotherapy

Endpoints	PFS is defined as the time from randomization to the first radiologically confirmed disease progression assessed by BIRC per RECIST 1.1 or death, whichever occurs first
Other intercurrent events and Handling Methods	New antineoplastic therapy: hypothetical policy (occurrence of such intercurrent events was censored)<br>Concomitant Medications/Treatments, Discontinuations, Clinical Progression: Treatment policy (Data collected and used for subsequent assessments regardless of intercurrent events)
Population-level Summary	Hazard ratio (HR)

2.3. Key Secondary ESTIMAND

2.3.1. Key Secondary Scientific Question of Interest

The key secondary clinical question of this study is overall survival improvement of suvemcitug plus chemotherapy compared to placebo plus chemotherapy regardless of the use of new antineoplastic therapy during treatment.

2.3.2. Key Secondary Estimand Attributes

Key secondary estimand attributes are presented in Table 2.3.1

Table 2.3.1 Critical Secondary Estimated Target Attributes

**Attribute**	**DESCRIPTION**
Population	Patients with recurrent epithelial ovarian cancer, fallopian tube cancer, and primary peritoneal cancer who met the inclusion and exclusion criteria and failed platinum-based chemotherapy
Treatment	Combination chemotherapy with suvemcitug versus placebo with subsequent antineoplastic therapy if needed
Endpoints	Time from randomization to subject death from any cause
Other intercurrent events and handling methods	New antitumor therapies: Treatment policy (Subsequent data will be collected and used regardless of intercurrent events)
Population-level Summary	Hazard ratio (HR)

2.4. Study Design

This study is a randomized, double-blind, multicenter, Phase III clinical study to compare the efficacy and safety, quality of life score, PK and immunogenicity of suvemcitug combined with chemotherapy (paclitaxel, liposomal doxorubicin, or

topotecan) and placebo combined with chemotherapy in patients with recurrent epithelial ovarian cancer, fallopian tube cancer, and primary peritoneal cancer who have failed platinum-based chemotherapy (platinum-resistant/refractory).

This study intends to enroll 411 subjects with recurrent epithelial ovarian, fallopian tube, and primary peritoneal cancer who have failed platinum-based chemotherapy. Subjects were screened, met the inclusion criteria and did not meet the exclusion criteria before entering the study. Enrolled subjects will be stratified according to the number of prior systemic therapies (1 or 2), whether they have platinum-refractory ovarian cancer, chemotherapy regimen (paclitaxel, liposomal doxorubicin, or topotecan), and whether they have received prior anti-angiogenic therapy and randomized in a 2:1 ratio to the following two arms:

Experimental arm: suvemcitug plus chemotherapy (paclitaxel, liposomal doxorubicin, or topotecan)

Control arm: Placebo + chemotherapy (paclitaxel, liposomal doxorubicin, or topotecan)

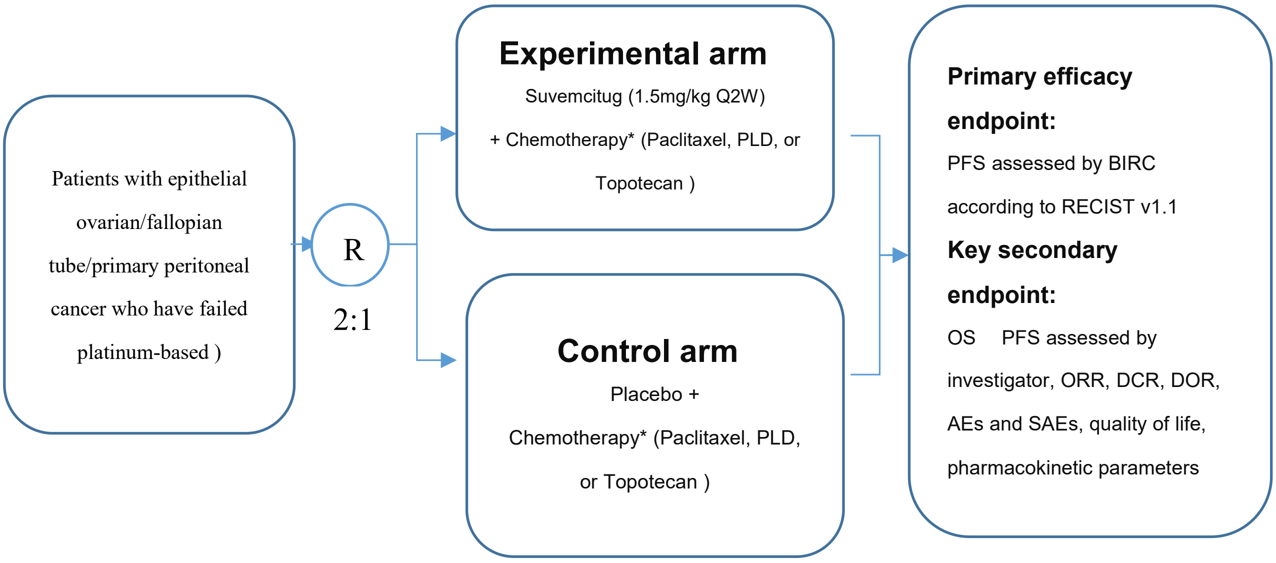

2.4.1. Study Plan and Design

Protocol Synopsis in Appendix 1. Study endpoints are listed in the protocol synopsis. Refer to the trial flow chart in Appendix 2 for additional information.

2.4.2. Randomization and Blinding

In this study, subjects who meet the inclusion criteria will be assigned to the treatment group and the control group using a dynamic randomization method according to the following stratification factors:

1) Number of types of previous systemic therapy (1 or 2);

2) Patients with platinum-refractory ovarian cancer;

3) Chemotherapy regimen (topotecan, liposomal doxorubicin, or paclitaxel);

4) Whether received previous anti-angiogenic therapy.

According to the randomization scheme, the Interactive Web Response System (IWRS) will assign a unique randomization number to subjects who meet the enrollment criteria according to the dynamic randomization rules and the enrollment sequence. Any randomized subject who withdraws from the clinical trial for any reason will retain the random number and no other subject is allowed to be reuse the random number, that is, no subject is allowed to participate in the study with the random number of the withdrawn subject, rather new random number will be used.

Double-blind techniques will be used in this study. Suvemcitug and vehicle will be packaged to maintain blinding. Drug blinding will be prepared by a person unrelated to this study. Neither the sponsor nor the representatives (except the IWRS service provider, etc.), nor the subjects nor the investigators involved in the study treatment medication or clinical evaluation of the subjects were aware of the group assignments.

For subjects who are inconsistent with the dose groups (treatment group, control group) in the actual medication process after randomization, for example, subjects who are identified as the treatment group by IWRS system, subjects who receive placebo during medication or subjects who are identified as the control group by IWRS system, and receive B D0801 during medication, it is agreed that in the safety analysis, the subject-level analysis depends on the dose group with the majority of medication times, that is, if more than half of the subjects belong to the treatment

group, the subjects will be classified as the treatment group for summary analysis at the time of analysis, otherwise they will be classified as the control group.

An in-house unblinded team will be established to conduct the 1st analysis. The unblinded team consisted of the sponsor 's unblinded medical science representative, statistician, statistical programmer, data management representative, pharmacovigilance representative, clinical pharmacology representative, regulatory affairs representative, quality management representative, and sponsor' s management representative. information, data, documents, etc. between the unblinded team and the blinded team were kept separate with a "firewall" (ie, electronic folder with account authority control) to avoid accidental unblinding. Specific job responsibilities and procedures are defined in the blinded maintenance operations manual. Investigators, subjects, and sponsor members in direct contact with the investigator or subject will remain blinded until the final unblinding of the study.

3. Sample Size Calculation

Approximately 411 subjects were planned to be randomized in this study and randomized with a 2:1 ratio to the treatment and control arms (274 and 137, respectively). A statistically significant improvement in at least one endpoint of PFS and OS was considered a success. The study is event-driven and the specific analysis time for each endpoint is determined by the number of events. In the Phase I study, the median progression-free survival (PFS) for platinum resistance was 5.9 months in the 1.5 mg/kg dose group of Suvemcitug. The AURELIA trial in recurrent ovarian cancer showed a median PFS of 6.7 months for suvemcitug combined with chemotherapy in patients with advanced platinum-resistant ovarian cancer [1] assuming a median PFS of 4.4 and 6 .4 months for the control and experimental arms, respectively.

The number of PFS events was calculated based on the following assumptions:

- PFS per RECIST 1.1 by BIRC

- Based on previous clinical studies and clinical need, assuming a median PFS of 4.4 and 6.4 months in the control and experimental arms, respectively, corresponding to a hazard ratio (HR) of 0.69
- One-sided significance level α = 0.025
- Annual dropout rate 15%
- Enrollment approximately 23 months

Based on these assumptions, an analysis of the PFS endpoint at the time 304 PFS events are observed (approximately 27 months after the first patient is enrolled) will provide approximately 87% power. At the PFS analysis, if statistical positivity for PFS is achieved, a one-sided alpha of 0.0001 will be assigned to the OS endpoint for the analysis of this OS.

For the OS assumption for the placebo arm, refer to the placebo arm median OS result of the bevacizumab AURELIA study, i.e. 13.3 months [1] ; for the OS assumption for the experimental arm, refer to the bevacizumab AURELIA study trial arm (chemotherapy + bevacizumab) median OS (16.6 months)[1] , apatinib APPROVE study trial arm (liposomal doxorubicin + apatinib) median OS (23.0 months) [2] , sorafenib TRIAS study trial arm (topotecan + sorafenib) median OS (17.1 months) [3] , and BD0801 ovarian cancer Phase Ib paclitaxel arm (paclitaxel plus BD0801) median OS (18.9 months) [4] , assuming 19 months.

The number of OS events at the final analysis was calculated based on the following assumptions:

- Assuming a median OS of 13.3 and 19 months in the control and experimental arms, respectively, corresponding to a hazard ratio HR of 0.7
- One-sided significance level α = 0.0249
- Annual dropout rate 5%
- Enrollment approximately 23 months

Based on these assumptions, an analysis of the OS endpoint at the time 278 OS events are observed (approximately 43 months after the first patient is enrolled) will provide approximately 80% power.

4. Analysis Set

4.1. Efficacy Analysis Set

Efficacy analyses will be based primarily on the Full Analysis Set (FAS). If necessary, Per-protocol Set (PPS) can be used as supportive supplement. The Full Analysis Set (FAS) will be based on the Intention-to-Treat (ITT) principle and will include all subjects randomized regardless of whether they received study treatment (suvemcitug or placebo or paclitaxel, liposomal doxorubicin, topotecan hydrochloride).

Per-protocol set (PPS) will include subjects in FAS who do not significantly deviate from the protocol. The PPS set is a subset of the FAS whose specific criteria and their population will be finalized at the time of data review. Efficacy analyses based on the PPS set could be performed to support the primary efficacy analysis if the number of subjects differed by more than 10% between the PPS set and the FAS set.

4.2. Safety Analysis Set

Safety analysis set (SS) will be used for safety analysis. Includes all subjects who received at least 1 study treatment (suvemcitug or placebo or paclitaxel, liposomal doxorubicin, topotecan hydrochloride) .

4.3. Efficacy Evaluable Analysis Set

Efficacy evaluable analysis set (EEAS), a subset of F AS, includes subjects in FAS who received at least one dose of study treatment (suvemcitug or placebo or paclitaxel, liposomal doxorubicin, topotecan hydrochloride) and had at least one postbaseline disease assessment or died due to disease progression prior to the first disease assessments.

4.4. Pharmacokinetic Analysis Set

Pharmacokinetic analysis will be based on the pharmacokinetic (PK) analysis set (PKS). The PK analysis set will include all randomized subjects who have received at least one dose of Suvemcitug and have at least one available PK sample for analysis.

4.5. Immunogenicity Analysis Set

Immunogenicity Analysis Set (I mmunogenicity Analysis Set, IAS), which includes all enrolled subjects who have received at least one dose of study drug and have samples for testing antibodies to suvemcitug.

5. Statistical Analysis Methods

5.1. General analytical provisions

This section is general analytical provisions and will be specified otherwise if there are special provisions.

5.1.1. Presentation of Results

The analysis results will be presented in tabular, graphical and tabular form. Laboratory values will be summarized and presented after conversion to SI units. Descriptive statistics were summarized for variables as needed:

- For continuous variables, the number of cases, mean, standard deviation, median, minimum, and maximum will be presented.
- For categorical variables, frequency and percentage will be presented.
- For time-to-event variables, Kaplan-Meier method will be used to estimate the survival function and estimate the median survival time and its 95% confidence interval, and survival curves will be plotted.

Scheduled and unscheduled visit data will be presented in listings by subject and visit time. Unscheduled visit data were not included in the pooled analysis, except where noted, but were included in the analysis for the worst and best values.

If missing data are imputed, the original values will be presented in the data listings.

The analyses specified in this document may be considered pre-defined and finalized prior to database lock. Post-hoc analyses will be identified in the clinical study report (CSR).

All analyses and data processing will be completed using SAS ® Version 9.4 or higher.

5.1.2. Data processing

5.1.2.1. Baseline data

For safety measures, baseline was defined as the last available valid measurement before (including the day of) the first use of study treatment. If study treatment had not been administered at the time of data cut-off or date was missing, the date of randomization was substituted.

For efficacy measures, baseline was defined as the last available valid measurement before (including the day of) randomization.

Unless otherwise specified, if the test value at an unscheduled visit meets the definition of baseline value, it must be confirmed that the test value occurred prior to the first use of study treatment (ie, the test date is prior to the first use of study treatment or, although the test date is the same as the first use of study treatment, the documented test time can prove that it occurred prior to the first use of study treatment).

If there were multiple tests on the same day that met the baseline definition and the test value was recorded at the specific time of measurement, the test value with the latest test time was used, and if the specific time was not recorded, the scheduled visit measurement value was used.

5.1.2.2. Abnormal values, missing data

Outliers will be identified and confirmed prior to final analysis. Every effort should be made to minimize the generation of missing data.

For time-to-event indicators in efficacy indicators, the missing data directly related to the primary efficacy endpoint, such as censoring, have been integrated into the treatment table of censoring rules. The missing or incomplete data not directly related to the primary efficacy endpoint, such as incomplete date of death, will be filled according to the conservative principle; for efficacy indicators (such as ORR, DCR), missing data will be regarded as invalid (non-responder); for quality of life indicators (EORTC QLQ-C30) in efficacy indicators, missing data will not be filled.

Missing dates may be imputed prior to being used for a calculation or comparison and are specified as follows:

- Disease history related (eg, date of initial diagnosis of disease, date of progression on front line therapy, etc.)
  - ✓ Missing information for 'Day' only: 'Day' was imputed as Day 15 of the month.
  - ✓ Missing "Day" and "Month" information simultaneously: If the year is earlier than the first medication date, fill in as June 30 of that year; if the year is consistent with the first medication date, fill in as January 1 of that year.
  - ✓ If the date is completely missing, no imputation will be performed.
- Start date (eg AE start date, concomitant medication start date)
  - ✓ Missing 'Day' information only: 'Day' was imputed as the first day of the month. If the imputed start date is used for a certain calculation or comparison (e.g. to judge treatment-emergent adverse events, prior medications, concomitant medications, etc.) and both the year and month are the same as the reference date for calculation or comparison (e.g. informed consent date, start medication date), the imputed start date is the reference date for calculation or comparison.
  - ✓ Missing information on "Day" and "Month" simultaneously: "Day" and "Month" were imputed as the first day of the first month of the year (i.e. 01 Jan). If the imputed start date is used for a calculation or comparison (e.g. to judge treatment-emergent adverse events, prior medications, concomitant medications, etc.) and the year is the same as the reference date used for calculation or comparison (e.g. informed consent date, start medication date), the imputed start date is the reference date used for calculation or comparison.

- ✓ If date is completely missing: if start date is imputed for a certain calculation or comparison (e.g., to judge treatment-emergent adverse events, prior medications, concomitant medications, etc.), then impute as reference date for calculation or comparison (e.g. informed consent date, start date).
- ✓ Imputation start date must not be later than end date.

- End date (e.g. AE resolution date, concomitant medication end date)
  - ✓ Missing 'Day' information only: Impute 'Day' information as the last day of the month.
  - ✓ Missing information on "day" and "month" simultaneously: "day" and "month" were imputed as the last day of the last month of that year (i.e. 31 December).
  - ✓ If date is completely missing: Impute as the end of study date for that subject.
  - ✓ Impute end date must not be earlier than start date. If a subject died and the imputed date was later than the date of death, the date of death was imputed.
- Date of start of new antineoplastic therapy
  - ✓ Missing 'Day' information only: Impute 'Day' information as the last day of the month.
  - ✓ Data imputation will not be performed if 'year' or 'month' is missing.
- Date of death
  - ✓ Missing information only on "day": impute with "1", impute date and known latest date + 1, take the maximum value.
  - ✓ If year or month is missing, no imputation will be performed.
- Date of clinical progression
  - ✓ Missing information on 'day' only: impute with '1'.
  - ✓ If year or month is missing, no imputation will be performed.

5.1.2.3. Time window

Unless otherwise specified, when summarizing data by visit, if there are multiple measurements within the analysis window for a visit, the measurement closest to the scheduled visit date will be used for analysis; if there are more than one measurement closest to the scheduled visit date, the measurement with the later measurement date will be used; and if there are multiple measurements within the same day, the last measurement on that day will be used. Each measurement will be listed in the listings.

5.2. Study Population Characteristics

5.2.1. Subject disposition

Subject disposition was summarized based on all screened subjects. Subjects who did not meet the inclusion and exclusion criteria, screening failure of subjects, termination of treatment of subjects, early withdrawal of subjects from the study, completion of subjects' trial, and randomization of subjects were summarized and listed in detail. Reasons for subjects excluding PPS and reasons for subjects excluding EEAS were tabulated based on all screened subjects.

5.2.2. Protocol Deviations

The number and percentage of subjects with important protocol deviations during the trial will be summarized based on the FAS analysis set, and reasons for important protocol deviations will be summarized and tabulated in detail by category. Subjects with multiple important protocol deviations in different categories will be counted in each corresponding category (by number). Subjects with important protocol violations or deviations requiring exclusion from the per protocol analysis set will be identified at the data review meeting prior to database lock.

5.2.3. Demographic and Baseline Disease Characteristics

Pooled analyses will be performed for demographic and baseline disease characteristics based on the FAS analysis set. Demographics included age at informed consent, age group ($\leq$ 65 years,>65 years), sex, childbearing potential (yes, no), ethnicity (Han, other), height, weight, body surface area, etc.

Baseline disease characteristics included ECOG score, tumor origin (epithelial ovarian cancer, fallopian tube cancer, peritoneal cancer), pathological tumor type (high-grade serous adenocarcinoma, endometrioid carcinoma, other), FIGO stage (Stage 1, Stage 2, Stage 3, Stage 4, unknown), platinum-free interval, and platinum-free interval grouping (<1 month, 1-3 months, ≥3 months), prior anticancer drug therapy (chemotherapy, P ARP inhibitors, antiangiogenic inhibitors, PD-1/PD-L1 inhibitors, antibody/peptide-conjugated drugs , etc.), number of prior lines of systemic anticancer drug therapy, number of prior lines of systemic anticancer drug therapy (1, 2, 3, ≥4), time from first diagnosis to randomization (years), first tumor diagnosis cell differentiation grade, metastases (present or absent), site of metastases (peritoneal, pelvic, etc.), CA-125 classification (≤2ULN, 2ULN-1000, >1000), baseline sum of target lesion diameters, baseline sum of target lesion diameters (<median, ≥median), presence of ascites, pleural effusion, etc. .

Pooled analyses will be performed for stratification factors collected via the IWRS, such as number of prior systemic therapies (1 or 2), patients with platinum-refractory ovarian cancer (yes or no), chemotherapy regimens (topotecan, liposomal doxorubicin, or paclitaxel), and prior antiangiogenic therapy (yes or no).

Important baseline disease characteristics (eg, tumor origin, pathological tissue type, baseline CA-125 classification, number of prior lines of systemic antineoplastic therapy, FIGO stage, platinum-free interval, presence of ascites at baseline, presence of pleural effusion at baseline, etc) will be tabulated.

5.2.4. Past Medical History, Prior Medications, and Prior (Non-Drug) Therapies

Prior medical history therapies will be coded using the most current version of the Medical Dictionary for Regulatory Activities (MedDRA) and summarized and tabulated in detail by System Organ Class (SOC) and Preferred Term (PT).

Prior medications will be coded using the latest version of the World Health Organization Drug Dictionary (WHO Drug) and will be summarized and listed in

detail by chemical subgroup and generic drug name. Prior medications will be summarized separately by anticancer and nonanticancer medications.

Prior radiotherapy, non-drug therapies, etc. were planned to be categorized and summarized, and listed in detail.

5.2.5. Drug exposure and compliance

Drug exposure analysis will be based on Safety Analysis Set (SS). Parameters for the pooled analysis of drug exposure were defined as follows:

- Duration of dosing (weeks) = (date of last dose – date of first dose + 1)/7.
- Intensity of drug exposure (mg/kg/week) = the ratio of the actual amount of drug administered per unit body weight of the subject to the total duration of dosing (weeks) of the subject, where the duration of dosing is rounded up after calculation according to (last dose date - first dose date + 1)/7.
- End of exposure: For subjects who discontinued treatment, end of exposure = last dose; otherwise end of exposure = data cut-off.
- Planned number of doses = (end of exposure – first dose + dosing interval)/dosing interval rounded down. Among them, BD0801 and placebo were administered 14 days apart, paclitaxel injection 7 days apart, doxorubicin hydrochloride liposome injection 28 days apart, and topotecan hydrochloride injection 3 times every 4 weeks for the first 3 weeks.
- Cumulative actual total dose administered = actual dose administered to the subject over the exposure time and is the sum of each dose administered.
- Cumulative planned dose = first planned dose × theoretical number of doses to be administered.
- First planned dose = Baseline weight/BSA × First planned dose unit.
- Relative dose intensity = cumulative actual total amount administered/cumulative planned total amount administered × 100%.

Descriptive statistics were used to summarize the total exposure, intensity of exposure, and relative dose intensity (%) of BD0801/placebo, paclitaxel, liposomal doxorubicin,

and topotecan hydrochloride received by subjects during treatment. Relative dose intensities were summarized by the following categories: < 80%, ≥ 80% to 100%, ≥ 1 00% to 120%, ≥ 120%, etc.

Duration of treatment (BD0801/placebo, paclitaxel, liposomal doxorubicin, topotecan hydrochloride) was summarized by actual group.

The number and percentage of subjects will also be summarized and tabulated by actual grouping for reasons of medication skipping, premature dosing, delayed dosing, dose change, permanent discontinuation, and infusion interruption during treatment.

5.2.6. Concomitant Medications and Concomitant Non-Drug Therapies

Concomitant medications included all drugs or vaccines received up to 28 days after the last dose of study drug (suvemcitug/placebo, topotecan, paclitaxel, liposomal doxorubicin) or initiation of new oncology therapy. Concomitant medications were coded using the latest World Health Organization Drug Dictionary (WHODrug) and summarized by therapeutic area (ATC classification ATC02), chemical subgroup (ATC classification ATC04), and generic drug name , and the frequency and percentage of subjects with concomitant medications were counted. Subjects were planned for a list of concomitant nondrug treatments.

5.2.7. Subsequent antineoplastic therapy

The number of subjects receiving subsequent anti-tumor therapy, subsequent systemic anti-tumor drug therapy, surgical therapy and radiotherapy will be summarized separately. Categories of subsequent antineoplastic agents received (eg, chemotherapy, PARP inhibitors, antiangiogenic agents, PD-1/PD-L1 inhibitors, and other antineoplastic agents) were summarized.

5.3. Efficacy analysis

Efficacy analyses will be based primarily on the Full Analysis Set (FAS), grouped by cohort assigned at randomization. Efficacy analyses based on the PPS set could be performed to support the primary analysis if the number of subjects differed by more than 10% between the PPS set and the FAS set.

5.3.1. Primary Efficacy Endpoint

5.3.1.1. Defined

The primary efficacy measure, PFS assessed by the BIRC according to RECIST 1.1, was defined as the time from randomization to the first radiologically confirmed disease progression assessed by the BIRC according to RECIST 1.1 or death, whichever came first.

5.3.1.2. Primary Estimate Objective and Master Estimate Method

Each attribute of the master estimate target is presented in Section 2.2. The study hypothesis is:

Null hypothesis H_0 : no difference in PFS between treatment arms v.s.

Alternative Hypothesis H_1 : PFS in the experimental arm is better than in the control arm

PFS assessed by BIRC according to RECIST 1.1 criteria will be descriptively statistically analyzed using Kaplan-Meier method and Brookmeyer-Crowley method will be provided with 95% confidence intervals for the primary efficacy measure and survival curves will be plotted.

Group comparisons will be performed using a stratified log-rank method stratified by the value recorded in the IWRS at randomization, including: number of prior systemic therapies (1 or 2), patients with platinum-refractory ovarian cancer (yes or no), chemotherapy regimen (topotecan, liposomal doxorubicin, or paclitaxel), and prior antiangiogenic therapy (yes or no). A stratified Cox regression model including the above randomization stratification factors was used to estimate the efficacy hazard ratio (HR) and 95% confidence interval between groups. If there are too few subjects or too few events in some strata to allow the model to converge, merging or removing some strata will be considered.

Intercurrent events and strategies are presented in Table 5.3.1

Table 5.3.1 Intercurrent Events and Handling Methods for PFS Endpoints

Intercurrent Events	Strategy	Handling Methods
New antineoplastic	Hypothetical	Occurrence of an intercurrent event,

therapy*	Strategy	treated as censored
Concomitant medication/treatment	Treatment policy Strategy	Subsequent assessments will be collected and used regardless of intercurrent events
Drug withdrawn	Treatment policy Strategy	Subsequent assessments will be collected and used regardless of intercurrent events
Clinical Progression	Treatment policy Strategy	Subsequent assessments will be collected and used regardless of intercurrent events

*: New antineoplastic therapy refers to new antineoplastic therapy administered prior to a PFS event.

Missing data and handling strategy are presented in Table 5.3.2

Table 5.3.2 Missing PFS endpoint data and handling measures

Missing Data	Handling measures
No valid tumor assessment at baseline or postbaseline	Censored: Censored on the day of randomization
Radiologically confirmed disease progression or death ≤ 1 missed disease assessment	Event: date of event is date of radiologically confirmed progression or death
Radiographically confirmed disease progression or death occurred after ≥ 2 consecutive missed disease assessments	Censored: censored at last disease assessment before ≥ 2 consecutive disease assessments missing
Lost to follow-up	Censored: censored at date of last disease assessment prior to lost to follow-up

PFS Censoring rules for are detailed in Appendix 3. Censoring will be summarized in the following categories: alive without PD, no baseline swelling assessment, no post-baseline valid swelling assessment, start of new antineoplastic therapy before event, PD or death after 2 or more consecutive missed swelling assessments, withdrawal of consent, and loss to follow-up as detailed in Appendix 4 .

5.3.1.3. Sensitivity Analysis

If applicable, the following sensitivity analyses will be performed, with the other aspects of each sensitivity analysis remain the same as the main analysis.

Sensitivity Analysis 1: In the primary analysis, radiographic disease progression or death occurring after ≥ 2 consecutive missed disease assessments were censored, and in order to assess whether it was non-informative, radiographic disease progression or death occurring after ≥ 2 consecutive missed disease assessments were treated as an event in the sensitivity analysis.

Sensitivity analysis 2: In order to assess HRs that were common within each stratum of stratification factors, unstratified log-rank methods were used to calculate p-values for between-group differences and unstratified Cox regression models were used to estimate between-group HRs for efficacy in sensitivity analyses.

Sensitivity analysis 3: To assess the impact of erroneous collection of stratification factors on the results, if the stratification factors collected in IWRS are quite different from the data collected in EDC, the *p* value of the difference between groups will be calculated using the stratified log-rank method using the data collected in EDC as the stratification factor, and the stratified Cox regression model will be used to estimate the HR for efficacy between groups.

Sensitivity Analysis 4: Differences between the two groups will be assessed using a non-proportional hazards model if data permit.

5.3.1.4. Supplemental Analysis

If applicable, the following supplemental analyses will be performed, with the other aspects of each supplemental analysis being the same as the main analysis.

Supplemental Analysis 1. To assess the improvement of progression-free survival with suvemcitug plus chemotherapy compared with placebo plus chemotherapy when subjects are allowed to use new antineoplastic therapy, the treatment policy strategy for new antineoplastic therapy will not be censored to assess the difference between the two groups.

Supplemental Analysis 2 . To assess the improvement in progression-free survival with suvemcitug plus chemotherapy compared to placebo plus chemotherapy in the

absence of major protocol deviations in compliance with protocol specifications, differences between the two arms were assessed based on the PPS.

Supplementary analysis 3. To assess the similarity of disease progression assessed by RECIST1.1 and clinical disease progression, clinical progression was analyzed using a composite variable strategy. At the time of analysis, the occurrence of clinical progression was also considered an event, with the date of event being the earliest of the investigator 's judgment of the date of clinical progression, date of death, and date of disease progression per RECIST 1.1 criteria assessed by BIRC.

5.3.1.5. Subgroup analysis

Subgroup analyses of the primary efficacy endpoint progression-free survival (assessed by BIRC using RECIST 1.1 criteria) will be performed for the following variables in this study.

- Baseline demographic characteristics (age group, ECOG score)
- Baseline disease characteristics: ascites (yes or no), number of prior lines of systemic antineoplastic therapy (1, 2, or ≥ 3), platinum-free interval (< 3 months or ≥ 3 months), sum of baseline target lesion diameters (< median or ≥ median), etc.
- Number of prior systemic therapies (1 or 2)
- Patients with platinum-refractory ovarian cancer (yes or no)
- Chemotherapy regimen (topotecan, liposomal doxorubicin, or paclitaxel)
- Prior antiangiogenic therapy (yes or no)

To assess PFS in each chemotherapy subgroup, subgroup analyses will be performed similarly to the main analysis. Given the small number of subjects stratified for subgroup analyses, unstratified log-rank method will be used to calculate p-values for differences between groups and unstratified Cox regression model will be used to estimate the HR for efficacy between groups, except for analyses of the 3 chemotherapy subgroups.

Forest plots will be produced to provide HRs and their 95% confidence intervals estimated from unstratified Cox regression models.

5.3.2. Key Secondary Efficacy Endpoints

5.3.2.1. Defined

OS is defined as the time from randomization to the date of death of a subject due to any cause; if no OS event occurs as of the data cutoff date, the date last determined to be alive before the data cutoff date will be censored.

5.3.2.2. Key Secondary Estimate Objectives and Estimate Methods

Key secondary estimation target attributes are described in Section 2.3.2. Intercurrent events and strategies are presented in Table 5.3.3 .

Table 5.3.3 Intercurrent Events and Handling method for OS Endpoints

Intercurrent Events	Strategy	DESCRIPTION
New antineoplastic therapy*	Treatment Policy strategy	Subsequent assessments will be collected and used regardless of intercurrent events

*: New antineoplastic therapy refers to new antineoplastic therapy administered prior to an OS event.

Missing data and handling method is presented in Table 5.3.4

Table 5.3.4 Missing data for OS endpoint and handling method

Missing Data	Handling measures
Lost to follow-up	Censored: censored date last confirmed alive prior to data cut-off date

The last confirmed survival date is the last known survival date and detailed algorithm is presented in Appendix 5.

Descriptive statistics will be performed using the Kaplan-Meier method and 95% confidence intervals will be provided using the Brookmeyer-Crowley method and survival curves will be plotted. Group comparisons will be performed using a stratified log-rank test stratified by the number of prior systemic therapies (1 or 2), patients with platinum-refractory ovarian cancer, chemotherapy regimens (topotecan, liposomal doxorubicin, or paclitaxel), and prior antiangiogenic therapy. A stratified Cox regression model including the above randomization stratification factors was used to estimate the efficacy hazard ratio (HR) and 95% confidence interval between

groups. If there are too few subjects or too few events in some strata to allow the model to converge, merging or removing some strata will be considered.

5.3.2.3. Sensitivity Analysis

Sensitivity analyses will be performed as follows, as applicable, with other aspects of each sensitivity analysis the same as the main analysis.

Sensitivity analysis 1 . In order to assess whether missing data due to loss to follow-up are independent of treatment and whether loss to follow-up impacts efficacy assessments, analyses will be performed after imputation of lost to follow-up data prior to survival analysis.

Sensitivity analysis 2 . To assess the hypothesis of common HRs within each stratum of the estimated objectives, unstratified log-rank methods were used to calculate p-values for between-group differences and unstratified Cox regression models were used to estimate between-group efficacy HRs.

Sensitivity analysis 3 . Differences between the 2 arms will be assessed using a non-proportional hazards model if data permit.

5.3.2.4. Supplemental Analysis

If applicable, the following supplemental analyses will be performed, with the other aspects of each supplemental analysis being the same as the main analysis.

Additional analysis 1 . To assess how long overall survival was prolonged with the use of suvemcitug plus chemotherapy compared to placebo plus chemotherapy in subjects without new antineoplastic therapy, a hypothetical strategy was censored for new antineoplastic therapy to assess the difference between the two arms.

Supplementary analysis 2. To assess the improvement of overall survival with suvemcitug combined with chemotherapy compared with placebo combined with chemotherapy in subjects without other anti-vascular anti-tumor therapies, rank preserving structural failure time models (RPSFTM, Rank Preserving Structural Failure Time Models) were used to assess the effect of other anti-vascular anti-tumor therapies on OS after the end of study treatment.

Supplementary analysis 3: To evaluate the improvement of overall survival with suvemcitug plus chemotherapy compared with placebo plus chemotherapy in the absence of major protocol deviations in compliance with the protocol, the difference between the two groups will be assessed based on the PPS.

Supplementary analysis 4: To evaluate the improvement of overall survival in the potential population BD0801 combined with chemotherapy compared with placebo combined with chemotherapy using anti-vascular therapy at the posterior line, anti-vascular therapy at the posterior line was considered as an intercurrent event, and the differences between the two groups were analyzed separately in the two subgroups of whether anti-vascular therapy was used at the posterior line.

5.3.2.5. Subgroup analysis

Subgroup analyses for OS will be performed in a manner similar to the primary endpoint .

5.3.3. PFS per RECIST 1.1 by Investigator

PFS per RECIST 1.1 by investigator is defined as the time from randomization to the first disease progression assessed by investigator per RECIST 1.1 or death, whichever occurs first. Similar analysis methods as for the primary efficacy endpoint will be used for analysis.

5.3.4. Investigator-assessed ORR and DCR per RECIST 1.1

ORR as assessed by investigator per RECIST 1.1 is defined as the proportion of subjects achieving CR and PR as assessed by investigator per RECIST 1.1. Point estimates and 95% confidence intervals (Clopper-Pearson exact method) will be calculated for ORR , and differences between groups and 95% confidence intervals will be assessed using Miettinen and Nurminen method with Cochran-Mantel-Haenszel weights, and stratification factors will be similar to the primary analysis using Cochran-Mantel-Haenszel test for computerized group comparisons.

5.3.5. Investigator-assessed DCR per RECIST 1.1

DCR as assessed by the investigator per RECIST 1.1 is defined as the proportion of subjects who achieve CR, PR, and SD as assessed by the investigator per RECIST 1.1. Analyses will be performed in a similar manner to ORR assessed by investigator according to RECIST 1.1 criteria.

5.3.6. ORR per RECIST 1.1 by BIRC

ORR per RECIST 1.1 criteria as defined by the BIRC as the proportion of subjects achieving CR and PR as assessed by the BIRC based on RECIST1.1. Analyses will be performed in a similar manner to ORR assessed by investigators according to RECIST 1.1 criteria.

5.3.7. DCR per RECIST 1.1 by BIRC

DCIR as assessed by the BIRC per RECIST 1.1 criteria is defined as the proportion of participants who achieve CR, PR, and SD as assessed by BIRC per RECIST1.1. Analyses will be performed in a similar manner to ORR assessed by investigators according to RECIST 1.1 criteria.

5.3.8. Investigator-assessed DOR per RECIST 1.1

DOR assessed by investigator according to RECIST 1.1 defined as first assessment of CR or PR to the first assessment of PD or death from any cause was assessed by the investigator according to RECIST 1.1. DOR was analyzed only in subjects who achieved CR or PR. Details of censoring rules for DORs are provided in Table 5.3.5 .

Table 5.3.5 DOR Censoring Rules

Missing/censored data	Handling strategy
No tumor assessment after BOR of PR or above	Censored: Censored at date of first CR or PR
Subject started new anticancer therapy	Censoring: censored at date of last disease assessment prior to new antineoplastic therapy or date of first PR or greater
Radiologically confirmed disease progression or death ≤ 1 missed disease assessment	Event: date of event is date of radiologically confirmed progression or death
Radiographically confirmed disease progression or death occurred after ≥ 2	Censored: censored at the date of last disease assessment or first occurrence of PR or better

consecutive missed disease assessments	before ≥ 2 consecutive disease assessments were missing
Lost to follow-up	Censoring: censored at date of last disease assessment prior to loss to follow-up or date of first PR or greater
Withdrew consent	Censored: censored at date of last disease assessment before withdrawal of consent or date of first PR or better

Descriptive statistics will be performed using the Kaplan-Meier method and 95% confidence intervals will be provided using the Brookmeyer-Crowley method and survival curves will be plotted.

5.3.9. DOR per RECIST 1.1 by BIRC

DOR assessed by BIRC according to RECIST 1.1 criteria is defined as the time from first assessment of CR or PR to first assessment of PD or death from any cause as assessed by BIRC according RECIST 1.1 criteria. Analyses will be performed in a similar manner to DOR assessed by the investigator according to RECIST 1.1 criteria.

5.3.10. Quality of Life Endpoints

Analyses of quality of life endpoint measures will be based on the FAS analysis set. Quality of life (QOL) evaluations in this study included participant EORTC QLQ-C30 and EORTC QLQ-OV28 functional scale scores. Protocol Versions 1.1 and 2.0 and above used the different translated EORTC QLQ-C30 scales and EORTC QLQ-OV28 scales. EORTC QLQ-C30 scale data will be combined and EORTC QLQ-OV28 scale will be analyzed separately according to the executive protocol version. Subgroups with V 2.0 were also analyzed for both baseline and postbaseline.

Data for the EORTC QLQ-C30 scale and EORTC QLQ-OV28 scale will be processed according to their respective manuals, such as linear transformation of raw scores into a range 0 to 100 by algorithm, etc. This is summarized by domain.

Scale completion rates, scores, change from baseline scores, and percentage change from baseline scores will be summarized by visit. It will include the score before the subject starts a new anticancer therapy, withdraws from the study, or dies.

Least squares means (LSMEANS) for change from baseline in quality of life score were calculated for each group based on a mixed-effect model for repeated measures as applicable, with the dependent variable of the mixed-effect model being change from baseline in quality of life score and the independent variable being baseline score, visit, treatment group, treatment by visit interaction, randomization stratification factor, etc. The model will include visits where at least 15% of subjects complete the score.

5.3.11. Multiplicity correction

To ensure an overall type 1 error control, a sequential testing approach is planned for PFS and OS. PFS was first tested for superiority at a one-sided alpha of 0.025 test level. If PFS is negative, no formal hypothesis testing will be performed for OS. If a positive result is reached for PFS, hypothesis testing will be performed for the OS endpoint. Given that the OS endpoint is immature at the time of PFS analysis, no formal statistical hypothesis testing will be performed for OS at the time of PFS analysis, but a one-sided alpha of 0.0001 will be assigned for this OS analysis; OS will be tested for superiority at the one-sided alpha of 0.0249 at the final analysis of OS (2nd analysis). The multiplicity control strategy is detailed in Figure 5.3.1

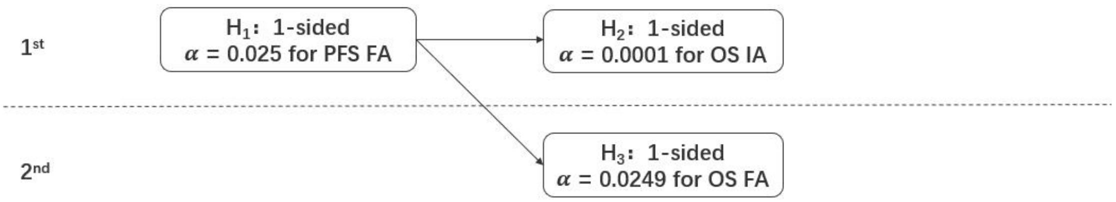

Figure 5.3.1 Multiplicity control strategy

5.4. Safety analysis

Safety analyses will be based on the SS analysis set. Safety evaluations included adverse events, physical examinations, vital signs, electrocardiograms (ECGs), and laboratory tests (hematology, blood chemistry, and urinalysis).

5.4.1. Adverse Events

All adverse events (AEs) will be coded according to the latest version of the International Conference on Harmonisation (ICH) Medical Dictionary for Regulatory Activities (MedDRA) and will include system organ class (SOC) and preferred term (PT). AEs were summarized by SOC and PT and sorted by decreasing overall number of subjects, unless otherwise specified. The National Cancer Center (NCI) Common Terminology Criteria for Adverse Events (CTCAE) version 5.0 was used to evaluate the severity of AEs.

Treatment Emergent Adverse Events (TEAEs) are defined as adverse events that occur after the first dose and up to 28 days after the last dose; or adverse events with incomplete onset dates and uncertainty whether an adverse event starts after the first dose; or an increase in toxicity grade present before the first dose but worsen after the first dose. Unless otherwise specified, only TEAEs were summarized. AEs with the preferred term 'disease progression' will not be included in the summaries. All AEs will be included in the listings, but TEAEs will be flagged. AEs with a preferred term of "disease progression" will be listed separately.

Other AE categories were defined as follows:

- Drug-related AEs: AEs judged as "definitely related", "probably related" or "possibly related" by the investigator recorded in the CRF, and judged as related if the relatedness was unknown;
- Serious Adverse Events (SAE): AEs marked as serious adverse events in the CRF;
- AEs leading to permanent discontinuation: AEs for which the action taken with drug in the CRF was' discontinued drug ';
- AEs leading to death: AEs with a final outcome of "death" in the CRF;

All AEs will be graded for severity using the National Cancer Institute (NCI) Common Terminology Criteria for Adverse Events (CTCAE) Version 5.0.

The number and incidence of AEs will be presented in the AE summaries and the AE incidence will be calculated based on the number of patients with AEs rather than the

number of patients with AEs. When calculating the number and incidence of AEs, a subject was counted as having multiple occurrences of the same AE. When summarizing the relatedness and severity of AEs, AEs with the strongest relatedness or the most severe relatedness were selected for summarization if they occurred in a single subject within the same SOC or PT.

The details of each AE/adverse reaction occurring in each subject shall be described in the listings, including type of AE, Me dDRA code, severity (CTCAE 1- Grade 5), start time/end time/duration, correlation of investigational drug or chemotherapeutic drugs, and outcome.

Descriptive statistics were performed for on-treatment AEs of special interest, such as hypertension and hypoesthesia. Special Concern AE categories are detailed in Appendix 6 .

An overall summary of the occurrence of T EAEs was prepared. Mainly includes: any TEAE, TRAE, any TESAE, any on-treatment ≥ Grade 3 SAE, TRSAE, any ≥ Grade 3 TEAE, study treatment-related ≥ Grade 3 TEAE, any TEAEs with an outcome of death, any TEAEs with a study-treatment-related outcome of death, any TEAEs leading to discontinuation of study treatment, study treatment-related TEAEs leading to discontinuation of study treatment, any TEAEs leading to discontinuation of study treatment, TEAEs leading to discontinuation of study treatment, any TEAEs leading to dose reduction of study treatment, TEAEs leading to dose reduction of each study treatment, on-treatment AESI , Study treatment-related on-treatment AESI.

TEAEs with an absolute difference in incidence of ≥2% between groups, TEAEs related to BD0801 or placebo with an absolute difference in incidence of ≥2% between groups, AEs with an incidence of ≥1% in either group during treatment related to any study treatment CTCAE grade ≥3, AEs with an incidence of ≥5% in either group during treatment, TRAEs with an incidence of ≥5% in either group during treatment, and AESIs with an incidence of ≥5% in either group during

treatment will be summarized. where the percentage threshold will be adjusted according to the actual data.

5.4.2. Laboratory tests

5.4.2.1. Handling of Laboratory Data

Laboratory results will be converted to SI units. For quantitative laboratory test results, if recorded as "< X", i.e., below the lower limit of detection, or recorded as "> X", i.e., above the upper limit of detection, the results were summarized as X and X, respectively, but the original results were presented in the listings. Numeric type results were graded using CTCAE version 5.0 or 4.3 and were graded based solely on laboratory test results (raw or derived data, or corrected data) regardless of the resulting intervention or symptoms (eg, hypokalemia grades 1 and 2 differ by presence or absence of symptoms and were therefore all classified as grade 1). If no corresponding term exists in this version, it is not graded. Indicator values such as serum sodium and serum potassium will be divided into high and low measures and graded by CTCAE in both directions.

CTCAE grades were adjusted as follows:

- Grade 5 refers to death, which cannot be judged only based on laboratory test values, so it will not appear in the grading summary;
- Missing values will be categorized as "missing values".

5.4.2.2. Abnormal liver laboratory values

Abnormal liver laboratory values were defined as any event that occurred during all on-treatment, postbaseline assessments (including scheduled and unscheduled values) in the following categories.

- Hy's law: alanine aminotransferase (ALT) and/or aspartate aminotransferase (AST) ≥ 3 × upper limit of normal (ULN), baseline alkaline phosphatase (ALP) ≤ ULN, total bilirubin > 2 × ULN
- AST: > 3 and ≤ 5 × ULN, > 5 and ≤ 8 × ULN, > 8 and ≤ 10 × ULN, > 10 and ≤ 20 × ULN, and > 20 × ULN; AST > 5 × ULN for more than 5 weeks.
- ALT: > 3 and ≤ 5 × ULN, > 5 and ≤ 8 × ULN, > 8 and ≤ 10 × ULN, > 10 and ≤ 20 × ULN, and > 20 × ULN; ALT > 5 × ULN for more than 5 weeks.

- Total bilirubin: > 1.5 × ULN and ≤ 2 × ULN, > 2 × ULN and ≤ 3 × ULN, > 3 × ULN and ≤ 10 × ULN, > 10 × ULN
- Alkaline phosphatase: > 1.5 × ULN

5.4.2.3. General Pooled Analysis

Descriptive statistics (including raw values and changes from baseline) and detailed listings of laboratory test item results completed at each scheduled visit were summarized by treatment group according to laboratory test results at baseline and each scheduled visit. If needed, a cross-classification table will be used to summarize changes in graded laboratory parameters from baseline to the most severe grade after dosing .

Laboratory values were classified according to the normal ranges provided by the study site: numeric results were presented as below the lower limit of the normal range, normal, and above the upper limit of the normal range, and grouped results were presented as normal, abnormal without clinical significance, and abnormal with clinical significance. Cross-classification tables from baseline were summarized for each scheduled visit. Pooled analyses were performed after calculating change from baseline and percent change from baseline for continuous measures.

Baseline C TCAE grade and postbaseline worst C TCAE grade were summarized for important hematology parameters (including decreased neutrophil count, increased total white blood cell count, etc.). Post-baseline data summaries will include both scheduled and unscheduled test results.

Cross-tabulations were made for qualitative baseline and postbaseline results (including negative, trace, 1 +, 2 +, 3 +, 4 +, etc.) for urine protein, urine glucose, and urine ketones;

Where required, numeric variables will be plotted in line graphs over time. All laboratory test results will be listed.

5.4.3. Vital signs

Descriptive statistics will be summarized and detailed listed for vital sign measurements (body temperature, systolic blood pressure, diastolic blood pressure, pulse, respiratory rate, etc.) and their changes from baseline at each visit.

Post-baseline vital sign abnormalities that meet the following established criteria will be summarized: pulse rate >100 beats/min, pulse rate <50 beats/min, systolic blood pressure ≥120-139 mmHg, systolic blood pressure ≥140-159 mmHg, systolic blood pressure ≥160 mmHg, diastolic blood pressure ≥80-89 mmHg, diastolic blood pressure ≥90-99 mmHg, diastolic blood pressure ≥100 mmHg, weight gain ≥5% - <10%, weight gain ≥10% - <20%, weight loss ≥5% - <10%, weight loss ≥10% - <20%, body weight decreased ≥20%, systolic blood pressure increased ≥20 mmHg from baseline, systolic blood pressure decreased ≥20 mmHg from baseline, diastolic blood pressure increased ≥15 mmHg from baseline, diastolic blood pressure decreased ≥15 mmHg from baseline.

5.4.4. ECOG score

Shift tables from baseline to the highest post-baseline score were summarized according to ECOG score at baseline and each visit.

ECOG scores will be listed for all subjects.

5.4.5. Physical examination

Results of physical examination items completed at each visit time point will be listed in detail.

5.4.6. ECG examination

Descriptive statistics will be summarized and detailed listed for ECG test results completed at each visit time point based on ECG test results (RR interval, PR interval, QRS interval, QT interval, QTcF interval, etc.) at baseline and each visit time point.

The number and proportion of subjects with QTcF intervals ≤ 450 msec, > 450 to 480 msec, > 480 to 500 msec, and > 500 msec will be summarized.

5.4.7. Echocardiography

Descriptive statistics will be summarized and detailed listed for "Left Ventricular Ejection Fraction" and other results completed at each visit time point according to echocardiography results at baseline and each visit time point.

5.4.8. Death

The total number of deaths during the trial, deaths within + 28 days after the last dose, and deaths after + 28 days after the last dose will be summarized and analyzed. Analysis of deaths included the total number of deaths and causes of death (adverse events, disease progression, other causes, etc.) in each actual group.

5.5. Analysis Time Point

The PFS endpoint was analyzed when approximately 304 PFS events were observed (approximately 27 months after the first patient was enrolled), and the first analysis was also performed for the OS endpoint. A second analysis of the OS endpoint was performed when approximately 278 OS events were observed (approximately 43 months after the first patient was enrolled). Primary and key secondary endpoint analysis time points are detailed in Table5.5 .1 .

Table5.5 .1 Primary and Key Secondary Endpoints

Analysis	Number of Events	Event Rate [1]	Estimated analysis time [2]
1st analysis	304 PFS events	74%	27 months
2nd analysis	278 OS events	68%	43 months

1: Event rate is the ratio of the number of events occurring and the overall number of subjects enrolled.
2: Time to first patient enrolled. This is estimated by the model and should be based on the actual time of occurrence.

5.6. Pharmacokinetic (PK) Analysis

Pharmacokinetic analysis will be based primarily on the PKS analysis set. Pharmacokinetic analysis applies to PK concentration analysis.

PK concentration analysis: PK concentration data will be summarized by tabular statistics according to each scheduled blood sampling time point as defined in the protocol, and PK profiles will be plotted.

Details of the population pharmacokinetic and exposure-response analyses will be described in a separate analysis plan and results will be reported independently of the CSR.

5.6.1. Pharmacokinetic Data Processing

5.6.1.1. Missing value

Missing values were replaced with 0 if they occurred before the first dose and not imputed if they occurred after the first dose.

5.6.1.2. Pharmacokinetic Data Processing

Data processing below the lowest limit of quantitation (BQL): When the P K concentration was below the lower limit of quantitation (BLQ), BLQ original recorded values were presented in listings. For statistical summaries, all concentration values below the lower limit of quantification will be handled as zero unless otherwise specified and will be presented as "BQL" in listings; plasma concentration data for BQL will be uniformly calculated as "0" when summarizing plasma concentrations at each blood collection point and plotting mean plasma concentration-time curves.

Handling of abnormal values of PK concentration: Abnormal values of PK concentration (such as abnormal values caused by protocol deviations, etc.) will be discussed and judged with the sponsor, site investigator and biological sample testing unit before database locking and the handling method will be determined. The reasons will be marked in the corresponding list or table and recorded.

5.6.1.3. Out of window data processing

Out-of-window plasma concentration data will not be included in the pooled analysis of individual serum drug concentrations, but will be flagged in the listings. For samples whose actual blood collection points did not exceed the window, the blood concentration at each blood collection point was descriptively described by the scheduled blood collection point.

5.6.2. Serum drug concentration analysis

The plasma concentration results at each time point after the first dose and multiple doses will be tabulated.

Descriptive summaries of plasma concentrations were provided for each blood collection point by scheduled blood collection time and included the number of cases, mean, standard deviation,% coefficient of variation, geometric mean, median, minimum, and maximum. Subject mean plasma concentration-time profiles and mean plasma concentration-time semilogarithmic profiles were also plotted. Standard deviation lines above the standard deviation of plasma concentrations for each scheduled blood sampling time point are shown in the figure.

5.7. Immunogenicity Analysis

Immunogenicity analysis was based on the I AS analysis set. Immunogenicity collected at different time points for subjects was listed, and if an immunopositive reaction occurred, the corresponding titer value was provided in the listing. Pooled analyses of baseline anti-drug antibody (ADA) positivity, treatment-induced anti-drug antibody positivity (TEADA), and treatment-induced duration of ADA positivity, neutralizing antibody (Nab) positivity were performed whenever possible.

ADA and neutralizing antibody (N a b) results will be listed and summarized by time after first dose. ADA listings will include ADA assay results, titers of ADA positive samples, and Nab assay results by individual patient and sample collection time point.

TEADA immune response can be divided into treatment-induced anti-drug antibody positive and treatment-enhanced anti-drug antibody positive. TEADA positive is defined as either:

- Treatment-induced anti-drug antibody positive: confirmed treatment-emergent ADA positive result with negative ADA baseline result;
- Treatment-boosted anti-drug antibody positive: positive ADA baseline result and post-treatment ADA titer value increased to ≥ 4-fold baseline titer value

Duration of treatment-emergent ADA positivity was defined as the length of time

from the patient 's first TEADA-positive sample to the patient' s last T E ADA-positive sample. Median duration of TEADA positivity will be reported and patients will be categorized into the following 2 categories based on their TEADA duration and listed separately:

- Transient TEADA:
  - TEADA was detected only at one sampling time point during treatment or follow-up observation (except at the last sampling time point and was considered persistent unless undetectable at a later time); or
  - TEADAs were detected at two or more sampling time points during the treatment period (including the follow-up period, if applicable), with the first and last TEADA-positive samples (regardless of any negative samples in between) separated by less than 16 weeks, and the subject 's last sampling time point being T E ADA-negative.
- Persistent TEADA:
  - TEADA detected at two or more sampling time points during treatment (including during follow-up, if applicable), where the first and last T E ADA-positive samples (regardless of any negative samples in between) are 16 weeks or longer apart; or
  - TEADA occurred only at the last sampling time point or less than 16 weeks prior to the last sample that was negative for TEADA.

The number of ADA and NAb positive samples will be summarized by nominal time after dosing. Anti-drug antibody positivity at baseline, treatment-emergent anti-drug antibody positivity (TEADA), and treatment-emergent ADA positivity duration will be summarized in tabular form.

5.8. Exploratory Analyses

Exploratory analyses will be performed to explore changes in subject CA-125 from baseline values and correlation with efficacy, as appropriate

- Analysis of trends in change from baseline in CA-125 versus change from baseline in sum of target lesion diameters was performed primarily.

- Line plots of change from baseline in CA125 by best response category subgroup.
- Kaplan-Meier curves of progression-free survival or overall survival by change from baseline in CA125 (< median, ≥ median).

If needed, PFS based on BIRC assessment, PFS based on investigator assessment, and overall survival will be analyzed after excluding subjects affected by the novel coronavirus outbreak from the FAS analysis set using the same analysis method as the respective primary analysis.

Exploratory analyses of the two subgroups of maximum percent reduction from baseline in CA125 levels (< median, ≥ median) were performed separately using similar methods as the subgroup analyses for the primary study endpoint.

5.9. Other analyses

Subject follow-up time was defined as the time from randomization to the last known date of survival or death. Inverse Kaplan-Meier method will be used to summarize the follow-up time of subjects in two groups.

Major protocol deviations, adverse events, etc. related to COVID-19 (Coronavirus disease 2019) will be summarized and analyzed. The number of subjects affected by COVID-19, the types of subjects affected (visit overall not done, visit overall out of window, visit part not done, visit part out of window, visit overall done in other ways, etc.), the time affected, death during the epidemic, TEAE ≥ Grade 3 during the epidemic, and subjects caused by the epidemic were summarized and analyzed from the exclusion of the analysis set. Pooled analyses were performed for the number of missed or delayed doses due to COVID-19. Analyses of BD0801/placebo and relative dose intensity of chemotherapeutic agents were planned for subjects before and after the outbreak. The occurrence of adverse events in each actual group was summarized according to system organ class and preferred term for treatment-emergent adverse events occurring during the epidemic.

Perform a list of situations impacted by COVID-19.

5.10. Data Monitoring Committee

An Independent Data Monitoring Committee (IDMC) will be established to ensure subject safety. I The DMC acts as an independent expert advisory group responsible for assessing the safety of the trial and ensuring the credibility of the trial. Enrollment in the trial, subject safety data will be reported at the meeting. Specific details regarding the IDMC can be found in the IDMC Charter.

The IDMC will periodically assess the progress and safety data of the trial and make recommendations to the sponsor to continue, suspend, modify, or terminate the study at the time of safety data review. Safety data review meetings will occur approximately every 6 months after the first subject is enrolled and will appropriately adjust the date of analysis if it is close to or overlaps with the protocol-specified date of the first analysis. Based on the discussion results of the planned review meeting, the IDMC may request an unplanned meeting if needed. The IDMC data review meeting involved unblinded clinical data and analysis results that were blinded to the sponsor.

6. Change from Protocol

Adverse Events

It is specified in the protocol that expected disease progression of the disease under study occurring during the study itself will not be recorded as an AE or SAE, and expected signs or symptoms resulting from disease progression will not be recorded as an AE or SAE. If the investigator considers disease progression related to the investigational product during the study, it should be recorded as AE or reported as SAE; the death caused by disease progression during the follow-up period at the end of treatment (28 days after the end of treatment) should be reported as SAE.

In the statistical analysis of this study, AEs with the preferred term "disease progression" will not be included in the planned summary of AEs, but A E with the preferred term "disease progression" will be tabulated.

PKS1, PKS2, and PKS3 Analysis Sets

The analysis sets for PKS1, PKS2, and PKS3 were defined in the protocol, and PKS1, PKS2, and PKS3 were not included in the definition of the analysis set for SAP given that this analysis did not include PK analysis for 3 chemotherapeutic agents (paclitaxel, liposomal doxorubicin, topotecan).

7. References

[1] Clinical Pharmacology and BioPharmaceutics Review (s) of Suvemcitug (Avastin ®) , FDA, 2005.

[2] Tiantian Wang, et al., Effect of Apatinib Plus Pegylated Liposomal Doxorubicin vs Pegylated Liposomal Doxorubicin Alone on Platinum-Resistant Recurrent Ovarian Cancer The Randomized VE Clinical Trial. JAMA Oncology, 2022 (8), 1169-1176.

[3] Radoslav Chekerov, et al., Sorafenib plus topotecan versus placebo plus topotecan for platinum-resistant ovarian cancer (TRIAS): a multicenter randomised, double-blind, placebo-controlled, phase 2 trial. 2018 (19), 1247-1258.

[4] Clinical Trial Summary Report of Safety, Tolerability, and Pharmacokinetics Trials of Suvemcitug in Combination with Chemotherapy in Chinese Patients with Platinum-Resistant Recurrent Epithelial Ovarian, Fallopian Tube, and Primary Peritoneal Cancer, 2022 Year 6 Month 20 .

8. APPENDICES

Appendix 1 Protocol Synopsis

**Title:**	**A Randomized Double-Blind Phase III Study of BD0801 (Suvemcitug) for Injection Combined with Chemotherapy versus Placebo Combined with Chemotherapy in Patients with Recurrent Epithelial Ovarian, Fallopian Tube, and Primary Peritoneal Cancer Who Have Failed Platinum-based Chemotherapy**
**Clinical Approval No.:**	2021LP00191, 2021LB00192
**Approved Date:**	09-Feb-2021, 14-May-2021
**Protocol No.:**	SIM-63-OC-301
**Protocol Version No.:**	V5.2
**Version Date:**	26 July 2023
**Leading site:**	Cancer Hospital CAMS
**Sponsor:**	Shanghai Xianxiang Medical Technology Co., Ltd.

Study title	A Randomized Double-Blind Phase III Study of BD0801 (Suvemcitug) for Injection Combined with Chemotherapy versus Placebo Combined with Chemotherapy in Patients with Recurrent Epithelial Ovarian, Fallopian Tube, and Primary Peritoneal Cancer Who Have Failed Platinum-based Chemotherapy
Protocol No.	SIM-63-OC-301
Sponsor	Shanghai Xianxiang Medical Technology Co., Ltd.
Study Phase	Phase III
Study subjects	Patients with recurrent epithelial ovarian, fallopian tube, and primary peritoneal cancer who have failed platinum-based chemotherapy
Study objectives	**Primary study objective**<br>• To evaluate the efficacy of suvemcitug plus chemotherapy versus placebo plus chemotherapy (paclitaxel, liposomal doxorubicin, or topotecan) in patients with recurrent epithelial ovarian, fallopian tube, and primary peritoneal cancer who have failed platinum-based chemotherapy.<br>**Secondary study objectives**<br>• To evaluate the safety of suvemcitug combined with chemotherapy versus placebo combined with chemotherapy (paclitaxel, liposomal doxorubicin, or topotecan) in patients with recurrent epithelial ovarian, fallopian tube, and primary peritoneal cancer who have failed platinum-based chemotherapy;<br>• To assess the effect of suvemcitug plus chemotherapy versus placebo plus

	chemotherapy (paclitaxel, liposomal doxorubicin, or topotecan) on quality of life in patients with recurrent epithelial ovarian, fallopian tube, and primary peritoneal cancer who have failed platinum-based chemotherapy;<br>• To assess the pharmacokinetic (PK) and immunogenicity (ADA) profiles of suvemcitug when administered in combination with chemotherapy (paclitaxel, liposomal doxorubicin, or topotecan) in patients with recurrent epithelial ovarian, fallopian tube, and primary peritoneal cancer who have failed platinum-based chemotherapy.<br>**Exploratory objectives**<br>• To explore the correlation between PK characteristics of suvemcitug and efficacy and safety<br>• To explore changes in CA-125 from baseline and correlation with efficacy in subjects
Study Endpoints	**Primary endpoint:**<br>**Blinded Independent Imaging Review Committee (BIRC) Progression Free Survival (PFS) per RECIST 1.1**<br>Defined as the time from randomization to the first radiologically confirmed disease progression assessed by BIRC per RECIST 1.1 or death, whichever came first;<br>**Secondary endpoints:**<br>**Overall survival (OS), key secondary endpoint**<br>Defined as the time from randomization to the date of death due to any cause;<br>**PFS per RECIST 1.1 by Investigator**<br>Defined as the time from randomization to the first radiologically confirmed disease progression assessed by the investigator per RECIST 1.1 or death, whichever came first;<br>**Incidence of adverse events (AEs) and serious adverse events (SAEs)**<br>Including any AE and SAE occurring from the subject 's signature of the informed consent form to 28 days after the last dose;<br>**Objective response rate (ORR) per RECIST 1.1 by investigator**<br>Defined as the proportion of subjects achieving complete response (CR) and partial response (PR) as assessed by the investigator based on RECIST 1.1;<br>**ORR per RECIST 1.1 by BIRC**<br>Defined as the proportion of subjects achieving CR and PR as assessed by BIRC based on RECIST 1.1;<br>**Disease control rate (DCR) according to RECIST 1.1 by investigator**<br>Defined as the proportion of subjects achieving CR, PR, and SD (stable disease) as assessed by the investigator based on RECIST 1.1;<br>**DCR per RECIST 1.1 by BIRC**<br>Defined as the proportion of subjects achieving CR, PR, and SD as assessed by BIRC based on RECIST 1.1;<br>**Duration of Response (DOR) per RECIST 1.1 by Investigator**<br>Defined as the time from the first assessment of CR or PR to the first assessment of

	progressive disease (PD) or death from any cause, as assessed by the investigator according to RECIST 1.1;<br>**DOR per RECIST 1.1 by BIRC**<br>Defined as the time from the first assessment of CR or PR to the first assessment of progressive disease (PD) or death from any cause as assessed by BIRC according to RECIST 1.1;<br>**Quality of Life Endpoints**<br>Change from baseline in EORTC QLQ-C30 and EORTC QLQ-OV28 functional scale scores (as assessed by the subject) after treatment;<br>**Pharmacokinetic evaluation**<br>Pharmacokinetic evaluation: Pharmacokinetic parameters in subjects receiving suvemcitug in combination with chemotherapy (paclitaxel, liposomal doxorubicin, or topotecan);<br>**Immunogenicity evaluation**<br>Immunogenicity evaluation: Immunogenicity response in subjects receiving suvemcitug in combination with chemotherapy (paclitaxel, liposomal doxorubicin, or topotecan).<br>**Exploratory Endpoints:** Correlation of suvemcitug PK profiles with efficacy and safety;<br>Change from baseline in CA-125 in subjects and correlation with efficacy.
Primary ESTIMAND	The main clinical question of this study is the progression-free survival improvement for suvemcitug plus chemotherapy compared to placebo plus chemotherapy, regardless of discontinuation during treatment, whether concomitant medication/therapy is used or not, assuming no new anti-tumor therapy is used.<br>The primary ESTIMAND consists of the following attributes:<br>• Population: patients with recurrent epithelial ovarian, fallopian tube, and primary peritoneal cancer who have failed platinum-based chemotherapy and meet the inclusion and exclusion criteria<br>• Treatment: suvemcitug plus chemotherapy vs placebo plus chemotherapy<br>• Endpoint: PFS, defined as the time from randomization to the first radiologically confirmed disease progression assessed by BIRC per RECIST 1.1 or death, whichever came first<br>• Intercurrent Events and Handling Method:<br>New antineoplastic therapy: hypothetical strategy (occurrence of such intercurrent events was censored)<br>Concomitant Medications/Treatments, Discontinuations, Clinical Progression: Treatment policy (Data collected and used for subsequent assessments regardless of intercurrent events)<br>• Population-level summary: Hazard ratio (HR)
Key Secondary Estimand	The key secondary clinical question of this study is overall survival improvement of suvemcitug plus chemotherapy compared to placebo plus chemotherapy regardless of the use of new antineoplastic therapy during treatment.

	Key secondary estimand, the estimand of overall survival, consist of the following attributes:<br>• Population: patients with recurrent epithelial ovarian, fallopian tube, and primary peritoneal cancer who have failed platinum-based chemotherapy and meet the inclusion and exclusion criteria<br>• Treatment: suvemcitug plus chemotherapy versus placebo plus chemotherapy with subsequent antineoplastic therapy if needed<br>• Endpoint: Time from randomization to subject death from any cause<br>• Intercurrent Events and Handling methods: New Antineoplastic Therapies, Treatment policy (Subsequent data will be collected and used regardless of intercurrent events)<br>• Population-level Summary: Hazard Ratio (HR)
Sample Size	It is estimated that the sample size of this study is 411 (274 in the treatment group and 137 in the control group). Considering actual screening waiting and other reasons, more patients may eventually be enrolled for no more than 10% of the estimated total sample size.
Study Design	This study is a randomized, double-blind, multicenter Phase III clinical study to compare the efficacy and safety, quality of life score, PK and immunogenicity of suvemcitug combined with chemotherapy (paclitaxel, liposomal doxorubicin or topotecan) and placebo combined with chemotherapy in patients withrecurrent epithelial ovarian cancer, fallopian tube cancer and primary peritoneal cancer who have failed platinum-based chemotherapy (platinum-resistant/refractory).<br>This study intends to enroll 411 subjects with recurrent epithelial ovarian, fallopian tube, and primary peritoneal cancer who have failed platinum-based chemotherapy. Subjects were screened, met the inclusion criteria and did not meet the exclusion criteria before entering the study. Enrolled subjects will be stratified according to the number of prior systemic therapy (1 or 2), whether they have platinum-refractory ovarian cancer, the planned combination chemotherapy regimen during the study (paclitaxel, liposomal doxorubicin, or topotecan), and whether they have received prior anti-angiogenic therapy and will be randomized in a 2:1 ratio to the following two arms for treatment:<br>Experimental arm: suvemcitug plus chemotherapy (paclitaxel, liposomal doxorubicin, or topotecan);<br>Control arm: placebo + chemotherapy (paclitaxel, liposomal doxorubicin, or topotecan);<br>The sponsor will close specific chemotherapy cohort enrollment at the appropriate time to avoid imbalance in chemotherapy arm proportion allocation.<br>Study treatment will continue until discontinuation criteria are met. Efficacy, safety, and subject quality of life will be assessed according to the visit procedures.<br>Follow-up for subsequent treatment information and survival information will continue after disease progression, every 3 months for subsequent treatment information and survival follow-up, and by telephone or other means until the subject meets the criteria for study withdrawal.

	The end of this study is defined as 18 months after the last subject is enrolled or after the number of events required for the primary study endpoint and key secondary endpoints of the study has been reached.<br>Subjects who were still receiving study treatment at the end of the study could continue receiving study treatment through additional extension studies, or other forms agreed with the sponsor, if they achieved stable disease or remission in efficacy evaluations and tolerated the study treatment, with approval from health authorities and ethics committees.
Investigational drug And Dosage and Administration	In this study, every 4 weeks (28 days) is a treatment cycle.<br>**Investigational product tested:**<br>**Suvemcitug was administered as:**<br>Suvemcitug, 1.5 mg/kg, IV infusion on Days 1 and 15 of each cycle.<br>**Placebo (Suvemcitug vehicle) was administered as:**<br>Placebo for Suvemcitug (Suvemcitug Vehicle), 1.5 mg/kg, IV infusion on Days 1 and 15 of each cycle.<br>**Non-investigational product tested:**<br>**Chemotherapy was administered as:**<br>Eligible subjects will use one of the following chemotherapy regimens at the investigator 's discretion and remain on chemotherapy for the duration of the study:<br>• Paclitaxel: 80 mg/m ^{2} administered once on Days 1, 8, 15, 22 of a 28-day treatment cycle.<br>• Liposomal doxorubicin: 40 mg/m ^{2} once on Day 1 of a 28-day treatment cycle.<br>• Topotecan hydrochloride: 4 mg/m ^{2} administered once on Days 1, 8, and 15 of a 28-day treatment cycle.<br>On Days 1 and 15 of each treatment cycle, subjects received prior infusions of suvemcitug or placebo followed by chemotherapy, and their vital signs were closely monitored. Blinded infusion of suvemcitug or placebo and open-label infusion of chemotherapy.
Study evaluation	**Efficacy evaluation:**<br>In this study, imaging examination (enhanced CT/CT plain scan or MRI) was used for baseline confirmation and efficacy assessment. Criteria for assessment will be the Response Evaluation Criteria in Solid Tumors (RECIST 1.1 criteria).<br>Baseline tumor imaging was performed within 2 weeks prior to the first dose. Imaging was performed prior to informed consent, and within 4 weeks prior to the first dose, this imaging tumor assessment could replace the baseline imaging after agreement with the sponsor. Efficacy assessments (including those based on RECIST1.1 criteria) were performed every 8 weeks for 48 weeks from the first dose (and every 12 weeks thereafter). Imaging times should follow calendar days and should not be modified for treatment delays until IRC-assessed disease progression is reached, the subject starts a new anticancer therapy, death, or the subject withdraws from the study.<br>Following disease progression, subjects will continue to be followed for subsequent treatment information and survival information, every 3 months for subsequent treatment

	information and survival follow-up, and by telephone or other means until the subject meets the criteria for study withdrawal.<br>**CA-125:**<br>CA-125 should be assessed by the same laboratory for the same subject, and each site is required to submit normal CA-125 ranges for the laboratory in which it is used. CA-125 testing was not required after disease progression was assessed by a blinded independent imaging review committee (BIRC) according to RECIST 1.1 criteria.<br>**Safety evaluation:**<br>Safety was evaluated by adverse events (adverse events graded using NCI-CTCAE version 5.0), clinical safety laboratory tests, vital signs, physical examinations, and electrocardiograms, and adverse events were recorded from the time the subject signed the informed consent form until 28 days after the last dose (suvemcitug/placebo or chemotherapy). The investigator was allowed to perform additional tests if necessary.<br>**Quality of Life Assessments:**<br>Subjects' EORTC QLQ-C30 and EORTC QLQ-OV28 functional scale scores were assessed at baseline and at other efficacy assessment time points.<br>**Pharmacokinetic evaluation:**<br>In the first treatment cycle, blood samples were collected within 30 min before the start of infusion for the first dose (Suvemcitug/placebo), 30 min after the end of infusion, 168 h (± 72 h) after the start of infusion for C1D15 and 30 min before the start of infusion for C1D15; in cycles 2 to 5, blood samples were collected within 30 min before the start of infusion for Day 1 of each cycle; and blood samples were collected at the end of treatment (within + 7 days) for pharmacokinetic evaluation.<br>**Immunogenicity evaluation:**<br>Blood samples were collected from subjects within 30 min before and 168 h (± 72 h) after the start of the first dose (Suvemcitug/placebo) infusion in Cycle 1, and within 30 min before and 28 days (± 5 days) after the start of the first dose (Suvemcitug/placebo) infusion in Cycle 3 for immunogenicity evaluation.
Subject Selection	**Criteria for inclusion:**<br>1. Age ≥ 18 years;<br>2. Histologically confirmed epithelial ovarian cancer, fallopian tube cancer or primary peritoneal cancer, pathological types are: high-grade serous adenocarcinoma, endometrioid carcinoma (G2 or G3), mixed epithelial carcinoma (high-grade serous adenocarcinoma and G2/G3 endometrioid carcinoma components must account for more than 50%), malignant Brunner 's tumor, undifferentiated carcinoma, dedifferentiated carcinoma and other rare types such as mesonephric duct-like carcinoma, gastric adenocarcinoma;<br>3. Patients with platinum-resistant recurrent ovarian cancer who have received a platinum-containing regimen and have progressed on platinum-containing regimen (platinum-refractory) or have had time to relapse from the end of platinum-containing therapy (at least 4 cycles) < 6 months (184 calendar days) during the platinum-containing regimen (first dose to 28 days after last dose).

	Definition of relapse or progression (any of the following):<br>A) Documented radiographic progression;<br>B) Persistent elevation of CA-125 (CA-125 ≥ 2 times the upper limit of normal and confirmed after 1 week) with clinical symptoms or physical examination suggestive of disease progression;<br>4. Progression during or after the most recent line of systemic therapy, or intolerable therapy, and at least one measurable lesion (assessed by investigator according to RECIST v1.1) within 4 weeks prior to randomization;<br>5. ECOG PS score of 0-1 within 7 days prior to the first dose;<br>6. Previous chemotherapy ended ≥ 3 weeks from the first dose of this study, monoclonal antibody anti-tumor therapy ended ≥ 4 weeks from the first dose of this study, and small molecule targeted therapy ended ≥ 2 weeks from the first dose of this study;<br>7. Treatment-related adverse events recovered to NCI-CTCAE Grade ≤ 1 (except Grade 2 alopecia);<br>8. Subjects must have adequate organ function and meet all of the following laboratory test results prior to enrollment:<br>A) Bone marrow (no blood transfusion or blood products, G-CSF or other hematopoietic stimulating factors were not used for correction within 14 days before blood routine examination during the screening period): neutrophils ≥ 1.5 × 10^9/L, hemoglobin ≥ 90 g/L, platelets ≥ 100 × 10^9/L;<br>B) Liver function: total bilirubin ≤ 1.5 × ULN, AST ≤ 3 × ULN, ALT ≤ 3 × ULN, alkaline phosphatase ≤ 3 × ULN; if liver metastasis, AST ≤ 5 × ULN, ALT ≤ 5 × ULN;<br>C) Renal function: serum creatinine ≤ 1.5 ULN, or creatinine clearance ≥ 60 mL/min calculated according to the Cockroft-Gault formula;<br>D) Coagulation function: INR ≤ 1.5 (INR range should be between 2-3 if the patient is receiving a stable dose of warfarin to manage venous thrombosis), APTT ≤ 1.5 ULN;<br>9. Estimated survival time ≥ 12 weeks;<br>10. For women of childbearing potential: agree to remain abstinent (avoid heterosexual intercourse) or use contraception with an annual failure rate of < 1% during treatment and for at least 6 months following the last dose of suvemcitug/placebo , paclitaxel, liposomal doxorubicin, or topotecan, whichever occurs later.<br>**Exclusion Criteria:**<br>1. Received > 1 line of systemic therapy for ovarian cancer following platinum resistance and/or > 1 line of non-platinum systemic therapy prior to platinum resistance.<br>2. Subjects who progressed during the first platinum-based chemotherapy (from first dose to 28 days after last dose);<br>3. Ovarian epithelial tumors with low malignant potential, such as low-grade serous adenocarcinoma, borderline tumors;<br>4. Mucinous carcinoma of the ovary, or clear cell carcinoma;<br>5. Non-epithelial tumors, such as sex cord and stromal tumors, germ cell tumors,

	carcinosarcoma, etc.;<br>6. Patients with other active malignant tumors within 5 years or at the same time (cured localized tumors, such as cutaneous basal cell carcinoma, cutaneous squamous cell carcinoma, cervical carcinoma in situ, etc. can be enrolled);<br>7. Any pelvic or abdominal radiotherapy;<br>8. Patient had recent major surgery or anticipated surgical intervention:<br>A) Major surgery or significant trauma within 28 days prior to enrollment;<br>B) Major surgical procedures anticipated during the course of the study, including but not limited to abdominal surgery (laparotomy or laparoscopy) prior to disease progression;<br>C) Open biopsy performed within 7 days prior to enrollment;<br>9. Known hereditary or acquired bleeding and thrombophilia (e.g., hemophilia, coagulopathy, thrombocytopenia, hypersplenism, etc.); clinically significant bleeding events, arterial or deep venous thromboembolic events, or superficial venous thrombosis and myenteric venous thrombosis requiring intervention within 6 months prior to enrollment;<br>10. Taking aspirin (> 325 mg/day) currently or recently (within 10 days prior to first dose);<br>11. Patients with a history of intestinal obstruction (including incomplete intestinal obstruction) within 3 months before enrollment; patients with a history of abdominal fistula, gastrointestinal perforation, abdominal abscess; patients with intestinal invasion found by imaging examination (CT examination, MRI examination) or pelvic examination during the screening period;<br>12. Severe infection requiring systemic antibiotic infusion or hospitalization during the screening period;<br>13. Patients with clinically manifested CNS (central nervous system) disease, or brain metastasis; stroke (CVA) or transient ischemic attack (TIA) within 6 months prior to enrollment;<br>14. Clinically significant cardiovascular disease:<br>A) Uncontrolled hypertension (defined as systolic blood pressure ≥ 150 mmHg and/or diastolic blood pressure ≥ 100 mmHg after drug treatment);<br>(2) History of myocardial infarction or unstable angina within 6 months prior to enrollment;<br>C) New York Heart Association (NYHA) class II and above heart failure;<br>D) Severe arrhythmia requiring medication; excluding asymptomatic atrial fibrillation with controlled ventricular rate;<br>15. Left ventricular ejection fraction < 50%;<br>16. Presence of neuropathy ≥ Grade 2 (CTCAE 5.0) at screening;<br>17. Presence of severe non-healing wound, ulcer or fracture; serous effusion (including pleural effusion and pericardial effusion) with clinical symptoms and requiring surgical treatment; ascites that is difficult to control;<br>18. Known serious hypersensitivity to the therapeutic agents or excipients used in the

	protocol;<br>19. Pregnant or lactating women;<br>20. Patients with proteinuria (urine protein > 1 + found in screening examination; or urine protein is 1 +, which fails to return to normal within 24 hours after retest);<br>21. Currently participating in another clinical study, or planning to start treatment in this study less than 30 days before the end of treatment in the previous clinical study;<br>22. Other conditions that, in the opinion of the investigator, would make participation in this study inappropriate.<br>23. Patients who have previously used BD0801
Criteria for Discontinuation of Subjects	Including but not limited to the following, the subject should discontinue the study treatment (including Suvemcitug/placebo and chemotherapy, if any of the following criteria are met):<br>1. Subject requests withdrawal or withdrawal of consent;<br>2. Radiographic disease progression assessed by the BIRC, unless the investigator judges that the subject is still benefiting from continued treatment;<br>3. Subject died;<br>4. Intolerable drug toxicity (the investigator may choose to terminate one of the agents in the regimen based on the type of AE and its relationship to different study drugs: Suvemcitug/placebo or chemotherapy, or both agents at the same time);<br>5. Poor compliance;<br>6. Sponsor decides to terminate the trial;<br>7. Pregnancy occurred during study treatment;<br>8. Others, such as unnecessary risks to the subject if the investigator continues to use the study treatment based on the subject 's medical condition or personal condition.
Criteria for Subject Withdrawal	Including, but not limited to, the subject should be withdrawn from the study and not followed up, whichever comes first:<br>1. Subject withdrew consent and refused further follow-up;<br>2. Subject lost to follow-up;<br>3. Subject died;<br>4. Termination of the study by the sponsor;<br>5. Other.
Statistical Methods	**Sample Size**<br>Approximately 411 subjects were planned to be randomized in this study and randomized in a 2:1 ratio to the treatment and control arms (274 and 137, respectively). Considering actual screening waiting and other reasons, it is possible to eventually enroll no more patients than 10% of the estimated total sample size .<br>The number of PFS events was calculated based on the following assumptions:<br>• PFS assessed by BIRC according to RECIST 1.1 followed an exponential distribution;<br>• According to previous clinical studies and clinical needs, assuming a median PFS of 4.4 and 6.4 months in the control and experimental arms, respectively, corresponding to a hazard ratio HR of 0.69;

	• One-sided significance levelα = 0.025<br>• Annual dropout rate 15%<br>• Enrollment approximately 23 months<br>Based on these assumptions, an analysis of the PFS endpoint at the time 304 PFS events are observed (approximately 27 months after the first patient is enrolled) will provide approximately 87% power.<br>• It is anticipated that OS data will not be mature at the time of the PFS analysis and no formal statistical hypothesis testing will be performed for OS at the time of the PFS analysis, but a one-sided alpha of 0.0001 will be assigned for this OS analysis.<br>The number of OS events at the final analysis was calculated based on the assumption that OS follows an exponential distribution;<br>• Assuming a median OS of 13.3 and 19 months in the control and experimental arms, respectively, corresponding to a hazard ratio HR of 0.7;<br>• One-sided significance levelα = 0.0249<br>• Annual dropout rate 5%<br>• Enrollment approximately 23 months<br>Based on these assumptions, an analysis of the OS endpoint at the time 278 OS events are observed (approximately 43 months after the first patient is enrolled) will provide approximately 80% power.<br>**Multiplicity correction**<br>To ensure an overall type 1 error control, a sequential testing approach is planned for PFS and OS. PFS was first tested for superiority at a one-sided α test level of 0.025. If PFS is negative, no formal hypothesis testing will be performed for OS. If a positive result is reached for PFS, hypothesis testing will be performed for the OS endpoint. α One-sided 0.0001 was assigned to the OS endpoint at the PFS analysis node for this OS analysis; OS was to be tested for superiority at the one-sidedα = 0.0249 test level at the time of the OS analysis (2nd analysis).<br>**Statistical Analysis Methods**<br>Efficacy analysis: Descriptive statistics using Kaplan-Meier method and Brookmeyer-Crowley method will be performed for primary efficacy endpoint PFS (assessed by BIRC according to RECIST 1.1 criteria) and 95% confidence intervals will be provided and survival curves will be plotted. Stratified log-rank test will be used to compare groups, and stratified Cox regression model will be used to estimate the efficacy hazard ratio (HR) and 95% confidence interval between groups. The time-to-event indicators in the secondary efficacy indicators were analyzed using the similar method to the primary efficacy indicator; the response rate indicators in the secondary efficacy indicators (such as ORR, DCR) were calculated using the point estimate and 95% confidence interval (Clopper-Pearson exact method), and the differences between groups were assessed using the Miettinen and Nurminen method; the quality of life indicators in the secondary efficacy indicators (such as EORTC QLQ-C30 and EORTC QLQ-OV28) were summarized by descriptive statistics.

	Safety analysis: Data for safety evaluation include adverse events observed during the trial and changes in laboratory test data before and after treatment. Descriptive statistics will be performed for adverse events. When necessary, the overall incidence of adverse events and the incidence of various adverse events will be compared between the two groups. In addition to the comparison of mean values before and after treatment, laboratory test data were mainly analyzed and listed in detail for cases that were normal before treatment but abnormal after treatment or abnormal before treatment but aggravated after treatment.<br>Pharmacokinetic analysis: Serum concentrations of Suvemcitug will be determined and summarized descriptively by group. If necessary, a population pharmacokinetic approach was planned to analyze the plasma concentration data, calculate the corresponding PK parameters, and explore the impact of PK on safety and efficacy.<br>Immunogenicity analysis: summarizes the frequency, rate, and duration of immunogenicity-positive reactions and corresponding titer values. If necessary, investigate the impact of immunogenicity on PK and safety.<br>**Analysis Time Point**<br>The first analysis was planned to occur when approximately 304 PFS events were observed in this study (approximately 27 months after the first subject was enrolled). A second analysis was performed when approximately 278 OS events were observed (approximately 43 months after the first subject was enrolled). Actual analysis time points are based on the number of events. If the PFS endpoint is positive in the first analysis and the OS endpoint shows a trend of benefit, the study will partially unblind the sponsor 's study team after data cleaning, conduct a comprehensive efficacy and safety analysis, and complete the regulatory submission. An in-house unblinded team will be established in this study to support the first analysis, and the specific workflow will be detailed in the Blinded Maintenance Operations Manual.

Appendix 2 TEST FLOW CHART

Project/Process	Screening Period		Treatment Period [20]					End of treatment Visit [20]	Survival Follow up
			Each cycle (every 28-day cycle)						
Visit Time / Day	D-27 to D0	D-6 to D0	D1 [21]	D8	D15	D22	D28	Within 28 days of end of treatment	Every 3 months
Window (Days)	-	-	1	± 3	± 3	± 3	± 3	-	± 7
Informed Consent	X								
Demographic data collection	X								
Medical History [1]	X								
Prior Medication	X								
Physical examination [2]		X	X	X	X	X	X	X	
Vital signs [3]		X	X	X	X	X	X	X	
ECOG score		X			X		X	X	
Blood pregnancy test [4, 22]		X					X [5]	X	
Coagulation [6, 22]		X			X		X	X	
Laboratory Safety tests [7, 22]		X		X [8]	X	X [8]	X	X	
12-lead Electrocardiogram [22]		X			X		X	X	
Echocardiography [9, 22]	X						X	X	
CA-125 [10, 22]		X	Performed every 8 weeks ± 7 days ≤ 48 weeks, > 48 weeks, every 12 weeks ± 7 days						

Project/Process		Screening Period		Treatment Period [20] Each cycle (every 28-day cycle)					End of treatment Visit [20]	Survival Follow up
Visit Time / Day		D-27 to D0	D-6 to D0	D1 [21]	D8	D15	D22	D28	Within 28 days of end of treatment	Every 3 months
Window (Days)		-	-	1	± 3	± 3	± 3	± 3	-	± 7
Imaging [11]		X		Performed every 8 weeks ± 7 days ≤ 48 weeks, > 48 weeks, every 12 weeks ± 7 days						
Efficacy Assessments [12]		X		Performed every 8 weeks ± 7 days ≤ 48 weeks, > 48 weeks, every 12 weeks ± 7 days						
Dosing – Suvemcitug or Placebo [13]				X [13]		X [13]				
Chemotherapy Administration [14]	Topotecan [15]			X [15]	X [15]	X [15]				
	Paclitaxel [16]			X [16]	X [16]	X [16]	X [16]			
	Liposomal doxorubicin [17]			X [17]						
Quality of life Assessments [18]		X		Performed every 8 weeks ± 7 days ≤ 48 weeks, > 48 weeks, every 12 weeks ± 7 days						
Pharmacokinetic Blood Sampling				Refer to the following table for PK blood sampling table and time window						
Immunogenicity				Refer to the following table for immunogenicity blood collection table and time window						

Project/Process	Screening Period		Treatment Period [20]					End of treatment Visit [20]	Survival Follow up
			Each cycle (every 28-day cycle)						
Visit Time / Day	D-27 to D0	D-6 to D0	D1 [21]	D8	D15	D22	D28	Within 28 days of end of treatment	Every 3 months
Window (Days)	-	-	1	± 3	± 3	± 3	± 3	-	± 7
Blood sampling									
Concomitant medication	X	X	X	X	X	X	X	X	
Adverse Events	X	X	X	X	X	X	X	X	
Survival Follow-up [19]									X

1. Medical history/surgical history: Including tumor diagnosis (including metastatic site), complications, allergic history and surgical history. Prior anti-tumor therapy: Previous anti-tumor therapy, including chemotherapy, radiotherapy, biological therapy, immunotherapy, endocrine therapy, etc. Including confirmation of histological diagnosis.
2. Physical examination included weight, height, skin, mucous membranes, head, neck, chest, abdomen, joints of the spine and extremities, and neurological examination. Height was measured at screening only. Further targeted physical examinations may be performed by the investigator based on symptoms. This test will only be done if chemotherapy is administered at a visit during the treatment period.
3. Blood pressure, pulse, respiratory rate, and body temperature should be measured within 15 minutes prior to each dose of suvemcitug/placebo; blood pressure and pulse should be measured at the end of each infusion of suvemcitug/placebo (+ 5 minutes) and 1 hour (± 10 minutes) after the end of each infusion of suvemcitug/placebo to prevent infusion reactions if the visit includes dosing (suvemcitug/placebo, topotecan, paclitaxel, liposomal doxorubicin) before the first dose. This test will be performed only if chemotherapy is administered at a visit during the treatment period.
4. Pregnancy testing is not required during testing in women without potential reproductive capacity. Women of non-childbearing potential were defined as those who had undergone surgery resulting in loss of fertility (eg, hysterectomy, bilateral oophorectomy, or bilateral salpingectomy), or were ≥ 60 years of age, or were ≥ 40 and < 60 years of age and had been amenorrheic for more than 12 months.
5. Performed during Cycle 2 and its subsequent even-numbered cycles.

6. Coagulation: prothrombin time (PT), activated partial thromboplastin time (APTT), thrombin time (TT), international normalized ratio (INR), if the dose included in the visit (suvemcitug/placebo, topotecan, paclitaxel, liposomal doxorubicin) should be administered before dosing.
7. Clinical laboratory safety tests, if included in the current visit (Suvemcitug/placebo, topotecan, paclitaxel, liposomal doxorubicin) should be performed prior to dosing, including:

- Blood routine: hemoglobin, red blood cell, total white blood cell, platelet, neutrophil, eosinophil, basophil, lymphocyte, monocyte.
- Blood biochemistry: total bilirubin, conjugated bilirubin, alkaline phosphatase (ALP), alanine aminotransferase (ALT), aspartate aminotransferase (AST), sodium, potassium, calcium, magnesium, chloride, phosphorus, albumin, glucose, total protein, cholesterol (total), triglyceride, creatinine, urea nitrogen (BUN) or urea, uric acid.
- Urine routine: urine protein, ketone body, glucose, red blood cells, white blood cells, if urine protein ≥ + 2, further 24-hour urine protein quantitative test is required.

8. Routine blood tests only, only if chemotherapy was administered at the visit.
9. Echocardiography, treatment with suvemcitug/placebo and liposomal doxorubicin should be withheld in subjects treated with liposomal doxorubicin (PLD) if LVEF is below normal (< 50%) or decreases by ≥ 15% from baseline values. LVEF assessments should be repeated within two weeks following continued treatment.
10. Throughout the study, it will be performed every 8 weeks ± 7 days at ≤ 48 weeks, > 48 weeks, and every 12 weeks ± 7 days until IRC-assessed disease progression is reached, the subject starts a new anticancer therapy, death, or the subject withdraws from the study.
11. Imaging was performed using CT or MRI for pelvic and abdominal and thoracic assessments. The investigator was also allowed to evaluate other sites in case of suspicion of metastases at other sites. Screening (baseline) imaging was performed within 2 weeks prior to the first dose and should also include brain assessments. If the subject had imaging prior to signing the informed consent and within 4 weeks prior to the first dose, the imaging tumor assessment may have replaced the baseline imaging upon agreement with the sponsor. Throughout the study, it will be performed every 8 weeks ± 7 days at ≤ 48 weeks, > 48 weeks, every 12 weeks ± 7 days, and using the same imaging method as used during screening. Imaging times should follow calendar days and should not be modified for treatment delays until IRC-assessed disease progression is reached, the subject starts a new anticancer therapy, death, or the subject withdraws from the study, whichever occurs first. The BIRC will perform an accelerated verification of disease progression when judged by the investigator as disease progression. If the investigator judges that the subject has experienced disease progression but has not been confirmed by the BIRC, the imaging assessment should continue to be performed according to the imaging assessment plan and imaging data should be submitted to the BIRC until disease progression is confirmed by the

BIRC.

12. Throughout the study, it will be performed every 8 weeks ± 7 days at ≤ 48 weeks, > 48 weeks, and every 12 weeks ± 7 days until IRC-assessed disease progression, start of new antineoplastic therapy by the subject, death, or withdrawal from the study.
13. Subjects were randomized within 24 hours prior to administration of Suvemcitug/placebo, topotecan, paclitaxel, liposomal doxorubicin on Cycle 1 Day 1 (C1D1). Subjects in the treatment and control arms of this study received blinded suvemcitug and placebo at 1.5 mg/kg. administered on Days 1 and 15 of a 28-day cycle, respectively, and were allowed to delay dosing for up to 3 days without toxicity, not to prematurely, and for up to 2 weeks in the event of unresolved toxicity. If suvemcitug (placebo) is administered on the same day as chemotherapy, infuse suvemcitug/placebo first. Infusion of chemotherapy agents should wait at least 1 hour after the end of the Suvemcitug/placebo infusion before starting. This study allows the investigator to perform dose modifications of suvemcitug/placebo (see Section 5.6 for dose modification scheme) and allows the investigator to continue to administer suvemcitug/placebo in the event of discontinuation of chemotherapy administration until the criteria for discontinuation of treatment are met.
14. Subjects received one of the paclitaxel, liposomal doxorubicin, topotecan chosen by the investigator for chemotherapy and remained unchanged during the treatment period of this study. Dose modifications for chemotherapeutic agents (paclitaxel, liposomal doxorubicin, topotecan) were allowed in this study. (See Section 5 .6 for dose modification scheme).
15. Topotecan dose: 4 mg/m ^{2} administered once on Days 1, 8, and 15 of a 28-day dosing cycle, with delays permitted up to 3 days and no early dosing in the absence of dosing toxicity.
16. Paclitaxel dose administered: 80 mg/m ^{2} administered once on Days 1, 8, 15, and 22 of a 28-day dosing cycle, with delays permitted up to 3 days and no early dosing in the absence of dosing toxicity.
17. Liposomal doxorubicin: 40 mg/m ^{2} once on Day 1 of a 28-day dosing cycle, with delays permitted up to 3 days and no early dosing in the absence of toxicity.
18. Subjects' EORTC QLQ-C30 and EORTC QLQ-OV28 functional scale scores were assessed at baseline and at other efficacy assessment time points.
19. When the subject continues to be followed for subsequent treatment information and survival information after disease progression, follow-up will be performed by telephone or other means until the subject meets the criteria for withdrawal from the study. Long-term survival follow-up will be performed every 3 months (12 weeks ± 7 days), mainly including the following items: (1) other anti-tumor treatment (treatment information related to ovarian cancer includes treatment name, means, start date, end date and time to progression) (2) survival. Survival and other anti-tumor status follow-up may be performed by telephone visit.
20. Physical examination, vital signs, coagulation function and laboratory safety test at the treatment visit may not be repeated if the interval from the previous examination does not exceed 3 days; physical examination, vital signs, ECOG score, coagulation function and laboratory safety test at the end of treatment visit

may not be repeated if the interval from the previous examination does not exceed 7 days; blood pregnancy test, 12-lead ECG and echocardiography may not be repeated if the interval from the previous examination does not exceed 1 4 days; concomitant medications and adverse events should be collected until 2 8 days after the end of treatment.

21. D1 window + 1 day is only applicable for Cycle 1, and ± 3 days for Cycle 2 and subsequent cycles.
22. Clinical routine tests completed locally prior to informed consent, if within the protocol-specified window for screening tests, may be used for screening assessments without repeat testing after agreement from the sponsor.

Appendix 3 PFS Censoring Rules

PFS is a time-to-event type endpoint and is influenced by intercurrent event handling strategies and missing data handling methods. Intercurrent events and missing data will be handled according to different analysis methods to form corresponding PFS censoring rule Table 8 .1 See for specific rules.

Appendix Table 8 .1 PFS Censoring Rules

Situation	Main analysis	Sensitivity Analysis 1	Supplemental Analysis 1	Supplemental Analysis 3
No baseline or postbaseline valid tumor assessments and no deaths	**Censored** : Censored on the day of randomization	Same as main analysis	Same as main analysis	Same as main analysis
New antineoplastic therapy started prior to event	**Censored** : Censored date of last disease assessment on or before date of randomization or start of new anticancer therapy	Same as main analysis	Treatment Policy Strategy, no impact on event judgment	**Censoring:** Date of last disease assessment on or before the date of randomization or start of new anticancer therapy is censored. (Event including clinical progression)
Occurred after 2 or more consecutive missed tumor assessments	**Censored** : Censored at date of last disease assessment prior to randomization or event	**Event** : Date of PD or death as event date	Same as main analysis	**Censored** : Censored on the date of last disease assessment prior to randomization or event. (Event including clinical progression)
No PD and no death	**Censored** : Censored at date of last disease assessment	Same as main analysis	Same as main analysis	**Censored** : censored at date of last disease assessment if no clinical

				progression<br>**Event** : date of clinical progression as event date if clinical progression occurred

Appendix 4 Reason for Censoring Category

Events and reasons for censoring will be summarized by treatment group. PFS censoring reasons will be categorized as follows:

- Survival without PD
- No baseline swollen assessment
- No post-baseline valid swelling score
- New antineoplastic therapy started prior to event
- PD or death after 2 or more consecutive missed assessments
- Withdrew consent: includes subjects alive without PD but withdrew consent
- Lost to follow-up: including subjects who remain without PD and withdraw from the study for reasons other than "withdrawal of informed consent"

Reasons for censoring for OS will be categorized at the time of final analysis for OS, such as:

- No death
- Withdrew consent: includes subjects who did not die but withdrew consent
- Lost to follow-up: including subjects who did not die, for reasons other than "withdrawal of consent"

Appendix 5 Last Known Survival Date

To obtain the last known survival date of the subject, the date of all actual measurements will be investigated, such as:

- Date of survival status in long-term survival follow-up is "alive"
- Date of end of treatment or end of trial reason noncompliance, lost to follow-up, death, and other reason
- Date of examination of target, non-target and new lesions for tumor assessment
- Start date and end date of study treatment administration (including actual administration only)
- Dates of laboratory tests, vital signs, physical examinations, etc.
- Start date and end date of adverse event
- Date of start of new antineoplastic therapy

Appendix 6 Adverse events of special interest

Adverse events of special interest will include the following groupings:

- Reversible posterior leukoencephalopathy syndrome
- Wound healing complications (≥ Grade 3)
- Arterial thromboembolic events
- Venous thromboembolic events (≥ Grade 3)
- Bleeding events (non-CNS) (≥ Grade 3)
- Hemorrhagic events (CNS)
- Congestive heart failure (≥ Grade 3)
- Hypertension (≥ Grade 3)
- Proteinuria (≥ Grade 4)
- Fistula and abscess
- Febrile neutropenia
- Gastrointestinal perforation
- Peripheral neuropathy (≥ Grade 3)
