# Supplementary material for: Suvemcitug plus chemotherapy in women with platinum-resistant recurrent ovarian cancer: the SCORES randomized, double-blinded, phase 3 trial
Source: Nat Cancer. 2026 Jan 9;7(1):182–93. doi: 10.1038/s43018-025-01085-z (PMC12858396; doi:10.1038/s43018-025-01085-z)
Supplement: Supplementary file 2 — Reporting Summary [file 43018_2025_1085_MOESM2_ESM.pdf]

nature portfolio

Corresponding author(s): Lingying Wu

Last updated by author(s): Sep 2, 2025

Reporting Summary

Nature Portfolio wishes to improve the reproducibility of the work that we publish. This form provides structure for consistency and transparency in reporting. For further information on Nature Portfolio policies, see our Editorial Policies and the Editorial Policy Checklist.

Statistics

For all statistical analyses, confirm that the following items are present in the figure legend, table legend, main text, or Methods section.

n/a	Confirmed	
☐	☒	The exact sample size (*n*) for each experimental group/condition, given as a discrete number and unit of measurement
☒	☐	A statement on whether measurements were taken from distinct samples or whether the same sample was measured repeatedly
☐	☒	The statistical test(s) used AND whether they are one- or two-sided<br>*Only common tests should be described solely by name; describe more complex techniques in the Methods section.*
☐	☒	A description of all covariates tested
☐	☒	A description of any assumptions or corrections, such as tests of normality and adjustment for multiple comparisons
☐	☒	A full description of the statistical parameters including central tendency (e.g. means) or other basic estimates (e.g. regression coefficient) AND variation (e.g. standard deviation) or associated estimates of uncertainty (e.g. confidence intervals)
☐	☒	For null hypothesis testing, the test statistic (e.g. *F*, *t*, *r*) with confidence intervals, effect sizes, degrees of freedom and *P* value noted<br>*Give P values as exact values whenever suitable.*
☒	☐	For Bayesian analysis, information on the choice of priors and Markov chain Monte Carlo settings
☒	☐	For hierarchical and complex designs, identification of the appropriate level for tests and full reporting of outcomes
☒	☐	Estimates of effect sizes (e.g. Cohen's *d*, Pearson's *r*), indicating how they were calculated

Our web collection on statistics for biologists contains articles on many of the points above.

Software and code

Policy information about availability of computer code

Data collection	The EDC system of cubeCDMS® v1.1 from Shanghai CRScube Co., Ltd.; The IRT system of IRTON® v5.0 from Shanghai Shanhu Health Technology Co., Ltd.
Data analysis	SAS® 9.4 from SAS Institute.

For manuscripts utilizing custom algorithms or software that are central to the research but not yet described in published literature, software must be made available to editors and reviewers. We strongly encourage code deposition in a community repository (e.g. GitHub). See the Nature Portfolio guidelines for submitting code & software for further information.

Data

Policy information about availability of data

All manuscripts must include a data availability statement. This statement should provide the following information, where applicable:

- Accession codes, unique identifiers, or web links for publicly available datasets
- A description of any restrictions on data availability
- For clinical datasets or third party data, please ensure that the statement adheres to our policy

The trial protocol and statistical analysis plan are available in Supplementary Information. The deidentified individual data are available in Source Data files. All other data supporting the findings of this study (detailed AEs and Kaplan-Meier curves in the subgroup analyses) are available from the corresponding author on reasonable request.

Research involving human participants, their data, or biological material

Policy information about studies with human participants or human data. See also policy information about sex, gender (identity/presentation), and sexual orientation and race, ethnicity and racism.

Reporting on sex and gender	Adult women
Reporting on race, ethnicity, or other socially relevant groupings	Chinese
Population characteristics	Women with histologically-confirmed ovarian cancer that had progressed during or within 6 months after completing platinum-based therapy
Recruitment	At both outpatient and inpatient departments
Ethics oversight	Institutional review board at all participating centres

Note that full information on the approval of the study protocol must also be provided in the manuscript.

Field-specific reporting

Please select the one below that is the best fit for your research. If you are not sure, read the appropriate sections before making your selection.

☒ Life sciences ☐ Behavioural & social sciences ☐ Ecological, evolutionary & environmental sciences

For a reference copy of the document with all sections, see nature.com/documents/nr-reporting-summary-flat.pdf

Life sciences study design

All studies must disclose on these points even when the disclosure is negative.

Sample size	The planned sample size was 411. The statistical power was based on the total number events of PFS per RECIST, version 1.1 by BIRC and OS. Assuming a treatment effect (hazard ratio [HR]) of 0.69, corresponding to an improvement in median PFS from 4.4 months in the placebo plus chemotherapy arm, hereafter referred to as the placebo arm, to 6.4 months in the suvemcitug plus chemotherapy arm, hereafter referred to as the suvemcitug arm, 304 PFS events would provide about 87% power to detect the PFS treatment effect at one-sided significant level of 0.025.
Data exclusions	No data were excluded from analysis.
Replication	Not applicable.
Randomization	Eligible patients were randomized at a 2:1 ratio.
Blinding	Both the patients and outcome assessors were blinded.

Reporting for specific materials, systems and methods

We require information from authors about some types of materials, experimental systems and methods used in many studies. Here, indicate whether each material, system or method listed is relevant to your study. If you are not sure if a list item applies to your research, read the appropriate section before selecting a response.

Materials & experimental systems

n/a	Involved in the study	
☒	☐	Antibodies
☒	☐	Eukaryotic cell lines
☒	☐	Palaeontology and archaeology
☒	☐	Animals and other organisms
☐	☒	Clinical data
☒	☐	Dual use research of concern
☒	☐	Plants

Methods

n/a	Involved in the study	
☒	☐	ChIP-seq
☒	☐	Flow cytometry
☒	☐	MRI-based neuroimaging

Clinical data

Policy information about clinical studies

All manuscripts should comply with the ICMJE guidelines for publication of clinical research and a completed CONSORT checklist must be included with all submissions.

Clinical trial registration	ClinicalTrials.gov Identifier: NCT04908787
Study protocol	Uploaded as supplementary document
Data collection	June 5, 2021 to Oct 11, 2024
Outcomes	The primary endpoint was progression-free survival as assessed by an independent review committee. The key secondary endpoint was overall survival. Other secondary endpoints included objective response rate, disease control rate, duration of response, quality of life (QoL), safety, pharmacokinetics of suvemcitug, and anti-suvemcitug antibodies.

Plants

Seed stocks	Not applicable
Novel plant genotypes	Not applicable
Authentication	Not applicable
